# *In vitro* and *in vivo* anti-inflammatory active copper(II)-lawsone complexes

**DOI:** 10.1371/journal.pone.0181822

**Published:** 2017-07-25

**Authors:** Ján Vančo, Zdeněk Trávníček, Jan Hošek, Pavel Suchý

**Affiliations:** 1 Department of Inorganic Chemistry & Regional Centre of Advanced Technologies and Materials, Faculty of Science, Palacký University in Olomouc, 17. listopadu 12, Olomouc, Czech Republic; 2 Department of Human Pharmacology and Toxicology, Faculty of Pharmacy, University of Veterinary and Pharmaceutical Sciences Brno, Palackého tř. 1946/1, Brno, Czech Republic; Aligarh Muslim University, INDIA

## Abstract

We report *in vitro* and *in vivo* anti-inflammatory activities of a series of copper(II)-lawsone complexes of the general composition [Cu(Law)_2_(L_N_)_x_(H_2_O)_(2-x)_]·*y*H_2_O; where HLaw = 2-hydroxy-1,4-naphthoquinone, *x* = 1 when L_N_ = pyridine (**1**) and 2-aminopyridine (**3**) and *x* = 2 when L_N_ = imidazole (**2**), 3-aminopyridine (**4**), 4-aminopyridine (**5**), 3-hydroxypyridine (**6**), and 3,5-dimethylpyrazole (**7**). The compounds were thoroughly characterized by physical techniques, including single crystal X-ray analysis of complex **2**. Some of the complexes showed the ability to suppress significantly the activation of nuclear factor *κ*B (NF-*κ*B) both by lipopolysaccharide (LPS) and TNF-alpha (complexes **3**–**7** at 100 nM level) in the similar manner as the reference drug prednisone (at 1 μM level). On the other hand, all the complexes **1**–**7** decreased significantly the levels of the secreted TNF-alpha after the LPS activation of THP-1 cells, thus showing the anti-inflammatory potential *via* both NF-*κ*B moderation and by other mechanisms, such as influence on TNF-alpha transcription and/or translation and/or secretion. In addition, a strong intracellular pro-oxidative effect of all the complexes has been found at 100 nM dose *in vitro*. The ability to suppress the inflammatory response, caused by the subcutaneous application of λ-carrageenan, has been determined by *in vivo* testing in hind-paw edema model on rats. The most active complexes **1**–**3** (applied in a dose corresponding to 40 μmol Cu/kg), diminished the formation of edema simalarly as the reference drug indomethacine (applied in 10 mg/kg dose). The overall effect of the complexes, dominantly **1**–**3**, shows similarity to anti-inflammatory drug benoxaprofen, known to induce intracellular pro-oxidative effects.

## Introduction

**Fig 1:**
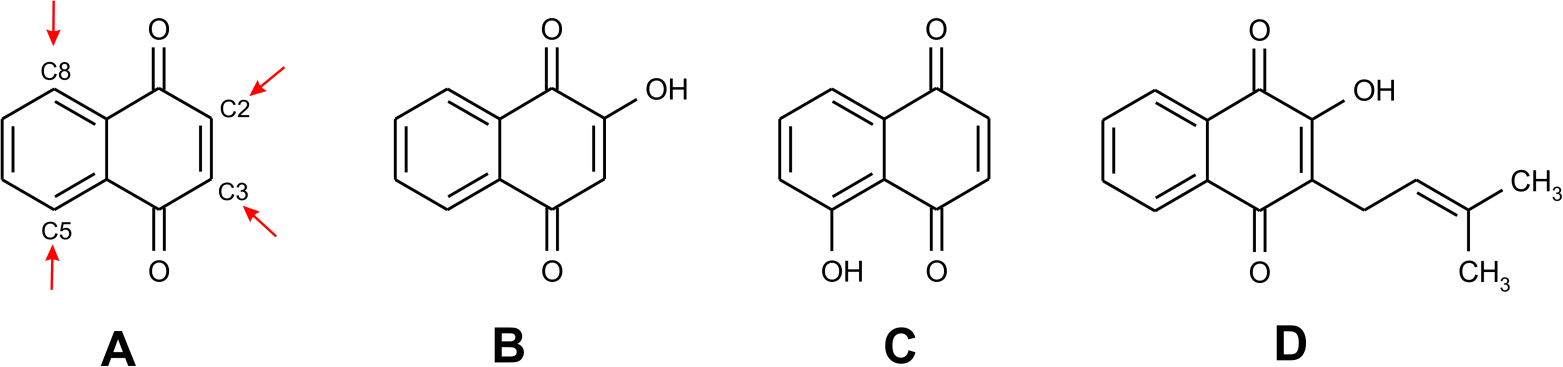
The general formula of 1,4-naphthoquinone showing most usual positions of substitutions, as indicated by arrows (A), and the formulas of selected 1,4-naphthoquinone derivatives isolated from natural sources: lawsone (B), juglone (C), and lapachol (D).

1,4-Naphthoquinones represent an important group of bioactive secondary metabolites of plants (see Fig 1 for the selected representatives isolated from natural sources) [1]. Depending on the substitution of the 1,4-naphthoquinone skeleton (mostly in positions 2-, 3-, 5-, and 8-), they show a variety of biological activities, including the antioxidant, anticancer, antimicrobial, anti-inflammatory, antimalarial and anti-HIV activities [2–5].

The hydroxyl-substitutions in the positions 2- (lawsone derivatives, HLaw) and 8- (juglone/plumbagin derivatives) open the possibility to utilize such 1,4-naphthoquinones as chelate ligands in transition metal complexes. To date, a few reports describing the syntheses and properties of copper(II), nickel(II), cobalt(II), chromium(III), iron(II), manganese(II), and zinc(II) complexes of 1,4-naphthoquinone derivatives with various compositions [6–8].

The reports dealing with the medicinal applications of the transition metal 1,4-naphthoquinone complexes are scarce. Recently, the interesting antimicrobial activity of transition metal complexes (Cu, Co, Fe, Ni, Cr) of 5-amino-8-hydroxy-1,4-naphthoquinone derivatives was reported [9]. In addition, the anticancer potential of transition metal complexes (M = Cu, Ni, Co, Mn) of lawsone, and complexes (M = Cu, Co, Ni) of juglone and lapachol were studied [7, 10, 11]. The copper(II), nickel(II), cobalt(II), and manganese(II) aqua-complexes involving lawsone with the general composition [M(Law)_2_(H_2_O)_2_] revealed interesting antiproliferative activities and the most active copper(II) complex showed the cytotoxicity against the RAW 264.7 cells, with IC_50_ = 2.5 μM. Very promising results of anticancer activity were found for copper(II), cobalt(II) and nickel(II) mixed-ligand complexes involving juglone (Hjug) or lapachol (Hlap) and 1,10-phenanthroline (phen) with the general composition [M(jug/lap)_2_(phen)] against human cervical carcinoma (HeLa), human liver hepatocellular carcinoma (HepG-2), and human colorectal adenocarcinoma (HT-29) cells, with quite low IC_50_ values in the range of 0.09–2.41 μM [10–11].

On the other hand, there are no known reports about the anti-inflammatory activity of transition metal complexes containing lawsone derivatives in contrast to the 1,4-naphthoquinone derivatives alone, and therefore we focused our attention towards the *in vitro* and *in vivo* studies of anti-inflammatory activity of the copper(II) compounds bearing the above-mentioned ligands. Moreover, our motivation is also connected with the fact that the present complexes were shown (based on the results of electrochemical studies) to possess the ability to take place in the production of reactive oxygen species (ROS) and to interact with DNA, as published in the previous paper [12], and thus, we wished to extend biological screening on these complexes with the aim to reveal any positive biological feature of these bioinorganic systems.

## Materials and methods

### Chemicals and materials

The starting chemicals Cu(CH_3_COO)_2_∙H_2_O, 2-hydroxy-1,4-naphthoquinone (lawsone, HLaw), imidazole (Im), 3,5-dimethylpyrazole (diMePz), pyridine (py) and its derivatives 2-,3-, and 4-aminopyridines (2-, 3-, and 4-apy) and 3-hydroxypyridine (3-OHpy), as well as all the solvents used, were purchased from Sigma-Aldrich (Prague, Czech Republic), Fischer-Scientific Co. (Pardubice, Czech Republic) and Acros Organics (Pardubice, Czech Republic), and were used without further purification.

The chemicals, media and methods used for the evaluation of biological activities were as follows: RPMI 1640 medium, phosphate-buffered saline (PBS) and a penicillin-streptomycin mixture were purchased from Biosera (Boussens, France). Fetal bovine serum (FBS) was obtained from HyClone (GE Healthcare, Logan, UT, USA). Phorbol myristate acetate (PMA), erythrosin B, *Escherichia coli* 0111:B4 lipopolysaccharide (LPS), dimethyl sulfoxide (DMSO) and *N*,*N*-dimethylformamide (DMF) for molecular biology, prednisone, dichlorofluorescein diacetate (DCFH-DA), and urethane were obtained from Sigma-Aldrich (Steinheim, Germany). The cytotoxicity against the THP-1 cell line, used for the *in vitro* evaluation of anti-inflammatory activities, was tested using a Cell Proliferation Reagent WST-1 kit from Roche Applied Science (Mannheim, Germany). The production of TNF-α was evaluated using a Human TNF-α Instant ELISA from eBioscience (Vienna, Austria). Quanti-Blue medium (Invivogen, San Diego, CA, USA) was used to detect reporter alkaline phosphatase. FLUOstar Omega microplate reader (BMG, Ortenberg, Germany) was employed to measure absorbance in the 96-well plates.

### Preparation of the complexes

**Fig 2:**
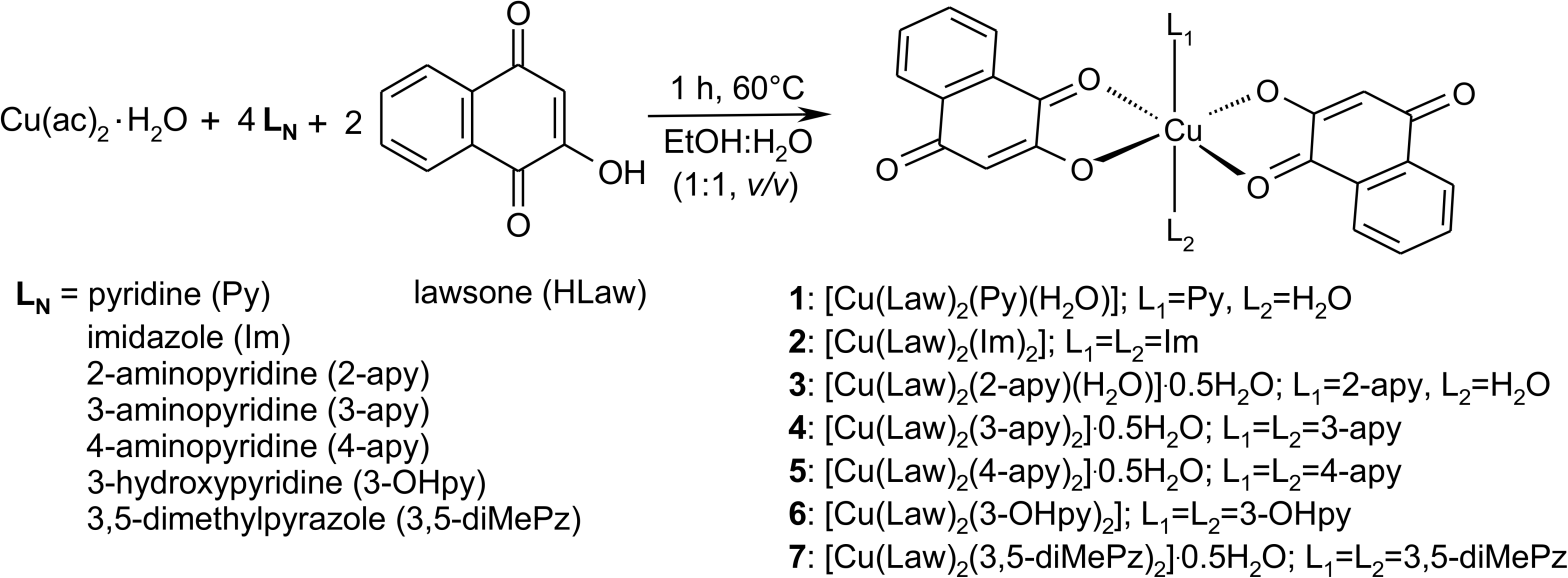
The general pathway of the preparation of complexes 1–7.

The copper(II) complexes **1**–**7**, having the general composition [Cu(Law)_2_(L_N_)_x_(H_2_O)_(2-x)_]·*y*H_2_O; where HLaw = 2-hydroxy-1,4-naphthoquinone, *y* = 0 or 0.5, and *x* = 1 when containing pyridine (**1**) and 2-aminopyridine (**3**), and *x* = 2 when containing imidazole (**2**), 3-aminopyridine (**4**), 4-aminopyridine (**5**), 3-hydroxypyridine (**6**), and 3,5-dimethylpyrazole (**7**) and as *N*-donor ligands (see Fig 2), were synthesized using a *one-pot* synthesis based on the reaction of the copper(II) acetate monohydrate with two equivalents of lawsone and 4-times excess of the appropriate *N*-donor ligand L_N_. The final reaction, containing a mixture of ethanol:water (1:1, v/v), was heated to 60°C and stirred vigorously for 1 h. After that, the products, which formed in a form of well-developed microcrystals, were isolated by vacuum filtration, washed with absolute ethanol (2 × 5 mL) and kept in desiccator over KOH for 2 days.

All the prepared complexes were characterized by elemental analysis, UV-Visible, FT-IR, electrospray-ionization mass spectrometry (ESI+ MS). Selected complexes were also analyzed by TG/DSC analyses, SQUID magnetometry and single crystal X-ray analysis.

For comparative purposes, the aqua-complex of the composition [Cu(Law)_2_(H_2_O)_2_]·0.5H_2_O was prepared by a modified method of S. Salunke-Gawali et al. [13]. The description of the preparative procedure and identification of the product is provided in S1 Text.

[Cu(Law)_2_(H_2_O)(py)] (**1**): Yield: 85%, Anal. calc for C_25_H_17_NO_7_Cu (*M*_r_ = 507.0): C, 59.2; H, 3.4; N, 2.8. Found: C, 58.9; H, 3.6; N, 2.8. ESI+MS (methanol, *m/z*): 160.0 (calc 160.0) [Cu^I^(H_2_O)(py)]^+^, 221.0 (calc. 221.0) [Cu^I^(py)_2_]^+^, 409.8 (calc. 409.9) [Cu(Law)_2_+H]^+^, 431.8 (calc. 431.9) [Cu(Law)_2_+Na]^+^, 644.7 (calc. 644.9) [Cu_2_(Law)_3_]^+^, 723.6 (calc. 723.9) [Cu_2_(Law)_3_(py)]^+^, 840.5 (calc. 840.9) [Cu_2_(Law)_4_+Na]^+^. IR (ν_ATR_/cm^-1^): 3230m, 2977m, 1655m, 1608m, 1585s, 1550s, 1442m, 1381s, 1338m, 1267s, 1250sh, 1211m, 1124m, 1065m, 983m, 833s, 811m, 782s, 733s, 700m, 666m. UV-Vis (Nujol): λ_max_ (nm) 491, 649sh, 791sh. UV-Vis (methanol): λ_max_ (nm) (ε_max_ × 10^3^ M^-1^ cm^-1^) 280 (29 570), 458 (4.81).

[Cu(Law)_2_(Im)_2_] (**2**): Yield: 92%, Anal. calc for C_26_H_18_N_4_O_6_Cu (*M*_r_ = 546.0): C, 57.2; H, 3.3; N, 10.3. Found: C, 57.3; H, 3.5; N, 10.3. ESI+MS (methanol, *m/z*): 149.0 (calc 149.0) [Cu^I^(H_2_O)(Im)]^+^, 199.0 (calc. 199.0) [Cu^I^(Im)_2_]^+^, 371.8 (calc. 372.0) [Cu(Law)_2_(Im)]^+^, 431.8 (calc. 431.9) [Cu(Law)_2_+Na]^+^, 644.6 (calc. 644.9) [Cu_2_(Law)_3_]^+^, 712.6 (calc. 713.0) [Cu_2_(Law)_3_(Im)]^+^. IR (ν_ATR_/cm^-1^): 3139m, 3074m, 2968m, 2878m, 1662m, 1609m, 1585m, 1542s, 1382m, 1340m, 1263s, 1251sh, 1212w, 1122w, 1071s, 984m, 833m, 784s, 733m, 657s. UV-Vis (Nujol): λ_max_ (nm) 502, 746sh. UV-Vis (methanol): λ_max_ (nm) (ε_max_ × 10^3^ M^-1^ cm^-1^) 280 (28.28), 464 (4.00).

[Cu(Law)_2_(H_2_O)(2-apy)]·0.5H_2_O (**3**): Yield: 90%, Anal. calc for C_25_H_19_N_2_O_7.5_Cu (*M*r = 531.0): C, 56.6; H, 3.6; N, 5.3. Found: C, 56.9; H, 3.9; N, 5.4. ESI+MS (methanol, *m/z*): 95.1 (calc 95.1) [2-apy+H]^+^, 175.0 (calc 175.0) [Law+H]^+^ and/or [Cu^I^(H_2_O)(2-apy)]^+^, 251.0 (calc. 251.0) [Cu^I^(2-apy)_2_]^+^, 329.9 (calc. 330.0) [Cu(Law)(2-apy)]^+^, 409.8 (calc. 409.9) [Cu(Law)_2_+H]^+^, 431.8 (calc. 431.9) [Cu(Law)_2_+Na]^+^, 644.6 (calc. 644.9) [Cu_2_(Law)_3_]^+^, 840.5 (calc. 840.9) [Cu_2_(Law)_4_+Na]^+^. IR (ν_ATR_/cm^-1^): 3454m, 3316m, 3196m, 1658m, 1640m, 1585m, 1550s, 1493s, 1448m, 1377m, 1340m, 1268s, 1250sh, 1214w, 1126w, 983m, 834m, 780s, 733s, 665m. UV-Vis (Nujol): λ_max_ (nm) 480, 672sh, 800sh. UV-Vis (methanol): λ_max_ (nm) (ε_max_ × 10^3^ M^-1^ cm^-1^) 280 (33.21), 460 (4.78).

[Cu(Law)_2_(3-apy)_2_]·0.5H_2_O (**4**): Yield: 82%, Anal. calc for C_30_H_23_N_2_O_6.5_Cu (*M*r = 607.1): C, 59.4; H, 3.9; N, 9.2. Found: C, 59.5; H, 4.2; N, 9.4. ESI+MS (methanol, *m/z*): 95.1 (calc 95.1) [3-apy+H]^+^, 175.0 (calc 175.0) [Law+H]^+^ and/or [Cu^I^(H_2_O)(3-apy)]^+^, 251.0 (calc. 251.0) [Cu^I^(3-apy)_2_]^+^, 423.9 (calc. 424.0) [Cu(Law)(3-apy)_2_]^+^, 431.8 (calc. 431.9) [Cu(Law)_2_+Na]^+^, 644.6 (calc. 644.9) [Cu_2_(Law)_3_]^+^, 738.6 (calc. 738.9) [Cu_2_(Law)_3_(3-apy)]^+^, 840.5 (calc. 840.9) [Cu_2_(Law)_4_+Na]^+^. IR (ν_ATR_/cm^-1^): 3407m, 3316m, 3213m, 3049w, 1652m, 1621s, 1585m, 1577m, 1548s, 1490m, 1448m, 1370m, 1330m, 1266s, 1255sh, 1217w, 1120m, 1056w, 985m, 899w, 848s, 781s, 736s, 697s, 667m. UV-Vis (Nujol): λ_max_ (nm) 472sh, 628, 742sh. UV-Vis (methanol): λ_max_ (nm) (ε_max_ × 10^3^ M^-1^ cm^-1^) 280 (30.42), 460 (4.25).

[Cu(Law)_2_(4-apy)_2_]·0.5H_2_O (**5**): Yield: 95%, Anal. calc for C_30_H_23_N_2_O_6.5_Cu (*M*r = 607.1): C, 59.4; H, 3.9; N, 9.2. Found: C, 59.4; H, 4.0; N, 9.1. ESI+MS (methanol, *m/z*): 95.1 (calc 95.1) [4-apy+H]^+^, 175.0 (calc 175.0) [Law+H]^+^ and/or [Cu^I^(H_2_O)(4-apy)]^+^, 251.0 (calc. 251.0) [Cu^I^(4-apy)_2_]^+^, 423.9 (calc. 424.0) [Cu(Law)(4-apy)_2_]^+^, 431.8 (calc. 431.9) [Cu(Law)_2_+Na]^+^, 644.6 (calc. 644.9) [Cu_2_(Law)_3_]^+^, 738.6 (calc. 738.9) [Cu_2_(Law)_3_(4-apy)]^+^. IR (ν_ATR_/cm^-1^): 3419m, 3333m, 3205m, 3071w, 1651m, 1622s, 1587s, 1547s, 1517s, 1455w, 1369m, 1327m, 1268s, 1247sh, 1210s, 1118w, 1060w, 1023m, 983m, 842m, 827s, 775s, 729s, 665m. UV-Vis (Nujol): λ_max_ (nm) 465, 673sh, 800sh. UV-Vis (methanol): λ_max_ (nm) (ε_max_ × 10^3^ M^-1^ cm^-1^) 276 (39.28), 466 (4.27).

[Cu(Law)_2_(3-OHpy)_2_] (**6**): Yield: 80%, Anal. calc for C_30_H_20_N_2_O_8_Cu (*M*r = 600.0): C, 60.1; H, 3.4; N, 4.7. Found: C, 59.9; H, 3.5; N, 4.6. ESI+MS (methanol, *m/z*): 96.1 (calc 96.1) [3-OHpy+H]^+^, 176.0 (calc 176.0) [Cu^I^(H_2_O)(3-OHpy)]^+^, 253.0 (calc. 253.0) [Cu^I^(3-OHpy)_2_]^+^, 330.9 (calc. 331.0) [Cu(Law)(3-OHpy)]^+^, 425.8 (calc. 426.0) [Cu(Law)(3-OHpy)_2_]^+^, 644.6 (calc. 644.9) [Cu_2_(Law)_3_]^+^, 739.6 (calc. 739.9) [Cu_2_(Law)_3_(3-OHpy)]^+^, 840.5 (calc. 840.9) [Cu_2_(Law)_4_+Na]^+^. IR (ν_ATR_/cm^-1^): 3117w, 3067w, 2908w, 2811w, 2753w, 2688w, 2630w, 2573w, 2525w, 2468w, 1657m, 1589m, 1568s, 1533s, 1479m, 1384m, 1341m, 1297m, 1269s, 1240m, 1215m, 1124m, 1109m, 1028w, 985m, 835m, 797m, 776m, 730s, 696s, 667w, 653m. UV-Vis (Nujol): λ_max_ (nm) 475sh, 742sh. UV-Vis (methanol): λ_max_ (nm) (ε_max_ × 10^3^ M^-1^ cm^-1^) 280 (34.31), 460 (4.53).

[Cu(Law)_2_(3,5diMePz)_2_]·0.5H_2_O (**7**): Yield: 80%, Anal. calc for C_30_H_27_N_4_O_6.5_Cu (*M*r = 611.1): C, 59.0; H, 4.5; N, 9.2. Found: C, 59.2; H, 4.7; N, 9.1. ESI+MS (methanol, *m/z*): 97.2 (calc. 97.1) [3,5diMePz+H]^+^, 177.0 (calc 177.0) [Cu^I^(H_2_O)(3,5diMePz)]^+^, 255.1 (calc. 255.0) [Cu^I^(3,5diMePz)_2_]^+^, 427.9 (calc. 428.0) [Cu(Law)(3,5diMePz)_2_]^+^, 644.6 (calc. 644.9) [Cu_2_(Law)_3_]^+^, 662.7 (calc. 662.9) [Cu_2_(Law)_3_+H_2_O]^+^, 740.6 (calc. 740.9) [Cu_2_(Law)_3_(3,5diMePz)]^+^. IR (ν_ATR_/cm^-1^): 3454w, 3207m, 3105w, 2924w, 1661m, 1607m, 1589s, 1578m, 1537s, 1475w, 1373m, 1330m, 1266s, 1247s, 1216w, 1116m, 1051w, 1026w, 984m, 839m, 781s, 733w, 660m. UV-Vis (Nujol): λ_max_ (nm) 468sh, 730. UV-Vis (methanol): λ_max_ (nm) (ε_max_ × 10^3^ M^-1^ cm^-1^) 280 (31.14), 458 (4.78).

### General methods for characterization

Elemental analysis (C, H, N) was performed on a Flash 2000 CHNS Elemental Analyser (Thermo Fisher Scientific, Waltham, USA). Infrared spectra were measured using a Nexus 670 FT-IR (Thermo Fisher Scientific, Waltham, USA) spectrometer using an ATR technique in the region of 650–4000 cm^-1^. The intensities of bands are defined as s = strong, m = medium and w = weak. Electrospray ionization mass spectra (ESI+MS) of methanol solutions of complexes **1**–**7** were acquired using an LCQ Fleet Ion Trap mass spectrometer (Thermo Fisher Scientific, Waltham, USA) in the positive ionization mode. Electronic spectra of complexes in methanol solutions were recorded on an HP 8453 UV-Visible spectrometer (Agilent Technologies, Santa Clara, USA). The selected complexes (**7** and the aqua-complex) were studied by thermal TG/DSC analysis in the range of 20–600°C with the linear thermal gradient of 5°C/min. The temperature dependence of magnetization was studied for the selected complexes **1**–**5** and the aqua-complex using a SQUID XL-7 magnetometer (Quantum Design, San Diego, USA) in the temperature range of 300–1.9 K.

### X-ray crystallography

Single crystal X-ray diffraction data of complex **2** were obtained on a Bruker D8 Quest diffractometer equipped with a Photon 100 CMOS detector, using the Mo-Kα radiation at 120(2) K. Data collection, data reduction, and cell parameters refinements were performed using the Bruker Apex III software package [14]. The molecular structure was solved by direct methods (SHELXS) and all non-hydrogen atoms were refined anisotropically on *F*^2^ using full-matrix least-squares procedure in SHELXL-2014 [15]. Hydrogen atoms were found in differential Fourier maps and their parameters were refined using a riding model. Molecular graphics were prepared and some structural features were evaluated and interpreted using Mercury, ver. 3.9. [16] The crystal data and structure refinements are given in Table 1.

**Table 1:** Crystal data and structure refinement for complex 2.

Complex	2
Empirical formula	C_26_H_18_CuN_4_O_6_
Formula weight (g·mol^-1^)	545.98
Temperature (K)	120(2)
Wavelength (Å)	0.71073
Crystal system	Orthorhombic
Space group	P*bcn*
a (Å)	13.778(3)
b (Å)	9.0229(18)
c (Å)	19.117(3)
α = β = γ (°)	90
V (Å^3^)	2376.5(8)
Z, D_calc_ (g cm^-3^)	4, 1.526
Absorption coefficient (mm^-1^)	0.970
Crystal size (mm)	0.18 × 0.16 × 0.16
F (000)	1116
θ range for data collection (°)	2.593 ≤ θ ≤ 27.529
Index ranges (*h*, *k*, *l*)	–16 ≤ *h* ≤ 17
	–11 ≤ *k* ≤ 11
	–24 ≤ *l* ≤ 24
Reflections collected	12734
Independent reflections	2728
Completeness to θ (%)	99.7
Data/restraints/parameters	2728/0/169
Goodness–of–fit on *F*^2^	1.011
Final R indices [*I*>2σ(*I*)]	*R*_1_ = 0.0551, w*R*_2_ = 0.1008
R indices (all data)	*R*_1_ = 0.1112, w*R*_2_ = 0.1200
Largest peak and hole (e Å^-3^)	0.849 and -0.603

### Anti-inflammatory activity testing *in vitro*

#### Maintenance and preparation of macrophages

The THP-1 human monocytic leukemia cell line was obtained from the European Collection of Cell Cultures (ECACC, Salisbury, UK) and THP1-XBlue™-MD2-CD14 cells were purchased from Invivogen (San Diego, USA). The cells were cultivated at 37°C in the RPMI 1640 medium supplemented with 2 mM L-glutamine, 10% FBS, 100 U/mL penicillin, and 100 μg/mL streptomycin in a humidified atmosphere containing 5% CO_2_. The culture was split twice a week when the cells had reached the concentration of 5–7×10^5^ cells/mL. The cell count and viability were determined following staining with erythrosin B. The cells were counted manually using a hemocytometer and a light microscope.

One hundred microliters of the stabilized THP-1 cells (5^th^-20^th^ passage) were split into 96-well plates to afford the concentration of 5×10^5^ cells/mL, and differentiation into macrophages was induced by the addition of phorbol myristate acetate (PMA), as described previously. [17] THP1-XBlue™-MD2-CD14 cells were used without any differentiation due to their stabile expression of MD2 and CD14 co-receptors.

#### Cytotoxicity assay

The tested compounds **1–7**, aqua-complex [Cu(Law)_2_(H_2_O)_2_]·0.5H_2_O, and lawsone were dissolved in DMF or PBS (due to low solubility, the concentration limit for most of the tested compounds in the culture media was 10 μM) and added to the THP-1 monocyte suspension in a culture medium in at least 5 concentration levels. The final concentration of the solvent in the culture medium was 0.1% (v/v). The cells were incubated at 37°C with 5% CO_2_ for 24 h. After the incubation, the cytotoxicity was determined by the Cell Proliferation Reagent WST-1 kit according to the manufacturer´s instructions. The IC_50_ values of the compounds were calculated from the obtained dose-viability curves, see S1 Fig.

#### Evaluation of TNF-α secretion

Differentiated THP-1 macrophages were pretreated with 100 nM solutions of compounds **1**–7, [Cu(Law)_2_(H_2_O)_2_]·0.5 H_2_O as well as the corresponding lawsone (HLaw) for 1 h. Whereas the complexes were dissolved in PBS, lawsone was dissolved in DMSO. With respect to the results of cytotoxicity assay, the concentration of 100 nM should not affect the viability of the target cells in any way. For comparative purposes, a conventional drug prednisone was used at the standard dose of 1 μM dissolved in DMSO. Then, the DMSO was added to the rest of the wells. The final concentration of DMSO reached the level of 0.1% in each well. Vehicle-treated cells contained a vehicle (0.1% DMSO) only.

The TNF-α secretion in the pretreated differentiated THP-1 macrophages was induced by the addition of 1 μg/mL LPS dissolved in sterile water. The basal cytokine expression level was determined in the control cells without the LPS stimulation. LPS triggers an inflammatory response through binding to toll-like receptor 4 (TLR-4) and subsequently activates the NF-*κ*B signaling pathway. The cultivation medium was aspirated 24 h after the LPS addition and the cell residue was removed by centrifugation. The concentration of the secreted TNF-α was determined using the Human TNF-α Instant ELISA kit.

#### Determination of NF-*κ*B activity

THP1-XBlue™-MD2-CD14 cells were transferred into a serum-free medium at the concentration of 5×10^5^ cells/mL. The floated cells were pretreated with the tested compounds (i.e. complexes **1–7**, [Cu(Law)_2_(H_2_O)_2_]·0.5H_2_O, and Hlaw, respectively) and stimulated either by LPS (1 μg/mL), or by the addition of human recombinant TNF-α (10 ng/mL; Invivogen), or by Pam3CSK4 (100 ng/mL; Invivogen), a synthetic triacylated lipoprotein and selective Toll-like receptor 1/2 agonist. After 24 h of the incubation, 20 μL of the cultivation medium was mixed with 180 μL of Quanti-Blue medium and incubated according to the manufacturer’s instructions at 37°C for 2 h. The activity of released alkaline phosphatase, directly proportional to the NF-*κ*B activity, was quantified from the difference in absorbance at 655 nm against the untreated control.

#### Evaluation of intracellular ROS production

To elucidate the possible pro/anti-oxidative effect of the tested complexes, a dichlorofluorescein method was used. [18–19] THP1-XBlue™-MD2-CD14 cells were seeded into a black plate in the concentration of 5×10^5^ cell/mL in serum-free medium and were incubated for 2 hours. In the following step, the solutions of the tested complexes, or a standard antioxidant Trolox^®^ were added at the final concentrations of 100 nM. The production of ROS was determined 1 and 24 h after the application of the compounds. 30 minutes before the determination of ROS production, DCFH-DA (5 μg/mL) dissolved in DMF was introduced into the cell medium. The intracellular fluorescence of the dichlorofluorescein product was measured by Fluostar Omega Microplate Reader (BMG Labtech) using λ(ex./em.) = 480/530 nm.

### Anti-inflammatory activity testing *in vivo*

This study was carried out in a strict accordance with the recommendations in the Guide for the Care and Use of Laboratory Animals of the National Institute of Health [20]. In addition, all the tests were conducted under the guidelines of the International Association for the Study of Pain. [21] The protocol was approved by the Expert Committee on the Protection of Animals Against Cruelty at the University of Veterinary and Pharmaceuticals Science in Brno (Permit Number: 35–2015). To minimize the suffering of laboratory animals, all pharmacological interventions were done under urethane anesthesia (applied 30 min prior the start of the experiment at the dose of 1.2 g/kg *i*.*p*.). The animals remained anesthetized during the whole duration of the experiment, until they were sacrificed by cervical dislocation. The anesthetized animals were placed onto the thermostated heating pad for preventing the loss of thermoregulation and their breathing and heart rate were checked regularly in about 10 min intervals. The animal tissues for *ex vivo* experiments were taken *post mortem*, immediately after all animals were sacrificed by cervical dislocation.

#### Animals

Wistar—SPF (6–8 weeks male) rats were obtained from the AnLab, Ltd., Prague. The animals were kept in plexiglass cages at the constant temperature of 22±1°C, and relative humidity of 55±5% for at least 1 week before the experiment. They were given food and water *ad libitum*. After a one-week adaptation period, male Wistar-SPF rats (200–250 g) were randomly assigned into ten groups (n = 7) of animals in the study. The first, control group, received 10% DMF (v/v in water, intraperitoneal; *i*.*p*.). The next eight groups were pretreated with complexes **1–7** and [Cu(Law)_2_(H_2_O)_2_]·0.5H_2_O (at the dose corresponding to 40 mmol Cu/kg; ca. 20 mg/kg) and involved into the carrageenan-treatment. The last group was treated with a non-steroidal anti-inflammatory drug Indomethacin (5 mg/kg), which served as a positive control (Indomethacin + carrageenan).

#### Carrageenan-induced hind paw edema evaluation and *ex vivo* histological evaluation

The carrageenan-induced hind paw edema model was used for the determination of the anti-inflammatory activity. [22] The animals were *i*.*p*. pretreated with the complexes **1–7**, or [Cu(Law)_2_(H_2_O)_2_]·0.5H_2_O or indomethacin (5 mg/kg; positive control) or 10% DMF (v/v in water for injections PhEur), 30 min prior to the injection of 1% λ-carrageenan (50 μL) into the plantar side of right hind paws of the rats. The paw volume was measured immediately after the carrageenan injection and during the next 6 h after the administration of the edematogenic agent using a plethysmometer (model 7159, Ugo Basile, Varese, Italy). The degree of swelling induced was evaluated as the percentage of change in the volume of the right hind paw after the carrageenan treatment from the volume of the right hind paw before the carrageenan treatment. These data were combined afterwards for all 7 animals within each experimental group and subjected to statistical evaluation by the one-way ANOVA with Bonferroni's multiple comparisons *post-hoc* test.

All the animals were sacrificed by cervical dislocation, and immediately after that, the affected hind paws were separated and underwent the process of dehydration and fixation, and were embedded into paraffin blocks by means of standard protocols. The histopathological changes, like infiltration of different skin elements and deeper laying tissues by the polymorphonuclear white blood cells (dominantly neutrophils and lymphocytes) stained by the standard hematoxylin/eosin staining, were evaluated.

### Statistical analysis

All the experiments were performed in triplicate, and the results are presented as mean values, with the error bars representing the standard error of the mean (SEM). A one-way ANOVA test was used for statistical analysis, followed by Bonferroni's multiple comparisons *post-hoc* test. A value of p < 0.05 was considered as statistically significant. GraphPad Prism 6.01 (GraphPad Software Inc., San Diego, CA, USA) was used to perform the analysis.

## Results and discussion

### Chemistry

The copper(II) complexes of the general composition [Cu(Law)_2_(L_N_)_x_(H_2_O)_(2-x)_]·*y*H_2_O; where HLaw = 2-hydroxy-1,4-naphthoquinone, *y* = 0 or 0.5, and *x* = 1 when containing pyridine (**1**) and 2-aminopyridine (**3**), and *x* = 2 when containing imidazole (**2**), 3-aminopyridine (**4**), 4-aminopyridine (**5**), 3-hydroxypyridine (**6**), and 3,5-dimethylpyrazole (**7**); and the aqua-complex [Cu(Law)_2_(H_2_O)_2_]·0.5H_2_O, where *x* = 0 and *y* = 0.5, were prepared as light to dark orange-brown crystalline solids. The complexes were characterized by elemental analysis, ESI+ mass spectrometry, FT-IR and UV-Visible spectroscopies, single crystal X-ray analysis (for complex **2**), SQUID magnetometry, and thermal (TG/DSC) analysis. The representative results of TG/DSC curves for complex **7** and the reference aqua-complex are presented in S2 and S3 Figs, respectively.

### Electrospray-ionization mass spectrometry (ESI+ MS)

The electrospray ionization mass spectra measured (ESI+ MS) in the positive ionization mode showed the presence of several peaks assignable to the species derived from the corresponding *N*-donor ligands (L_N_) [L_N_+H]^+^, the molecular ion of lawsone [Law+H]+ at *m/z* = 175.0 and furthermore also pseudomolecular species formed from the parent complexes **1**–**7**, *i*.*e*. [Cu^I^(L_N_)(H_2_O)]^+^; [Cu^I^(L_N_)_2_]^+^, [Cu(Law)(L_N_)_2_]^+^, [Cu(Law)_2_+H]^+^ at *m/z* = 409.8, [Cu(Law)_2_+Na]^+^ at *m/z* = 431.8, [Cu(Law)_2_(L_N_)]^+^, [Cu_2_(Law)_3_]^+^ at *m/z* = 644.6, and [Cu_2_(Law)_3_(L_N_)]^+^ species (see Experimental Section). From the mass spectral analysis, it is quite clear that the complexes undergo hydrolysis and ligand exchange reactions with the solvent molecules. The monodentate coordinated *N*-donor ligands seem to be the first to be involved in this type of reactions and thus, leaving the complex molecule. The representative mass spectra of complexes **1** and **3** are presented in S4 and S5 Figs.

### Infrared spectra

The IR spectra of complexes **1**–**7** (selected spectra for complexes **1** and **7** are depicted in S6 and S7 Figs) showed several characteristic vibrations belonging to the organic ligands [23]. Very intensive bands assignable to the stretching vibrations of the carbonyl groups ν(C^…^O) were detected at ca 1260 cm^-1^, while the bands observed in the region between 1640 cm^-1^ and 1450 cm^-1^ may be associated with the ν(C^…^C)_ring_, and ν(C^…^N) _ring_ vibrations. The bands observed at ca. 3139–2968 cm^-1^ can be associated with the ν(C–H)_arom_ vibrations. The complexes containing water molecules in the coordination sphere, or the water molecules of crystallization, or heterocyclic ligands containing hydroxyl, or amino groups (*i*.*e*. aminopyridines, and 3-hydroxypyridine) showed also the bands corresponding to ν(O–H) in the region of 3339–3207 cm^-1^, and to the ν(N–H) vibrations at 3454–3407 cm^-1^.

### UV-Visible spectra

The diffusion-reflection spectra showed intensive maxima (or intensive shoulder) in the region of 465–502 nm owing to the ligand-to-matal charge transfer (LMCT) transitions. Further, either one absorption band at 727–746 nm or two bands at 628–673 nm and 791–800 nm, owing to the *d-d* transitions of differently axially distorted octahedral polyhedron of Cu(II), have been identified. [24] The representative spectra of complexes **1** and **2** are shown in S8 and S9 Figs.

The solution spectra of the complexes, measured in methanol at 5×10^−4^ and 5×10^−5^ M concentrations (see S10 Fig), revealed intensive absorption bands at 276–280 nm and shoulders at ca. 300–330 nm, assignable to intraligand electron transitions within the 1,4-naphthoquinone and/or heterocyclic *N*-donor ligands, and one band assignable to CT transitions at higher wavelengths (at ca. 460–472 nm). The maxima of the first band appeared at 280 nm with ε_max_ ≈ 26 000–34 300 M^-1^ cm^-1^, except for complex **6**, with the maxima shifted to 276 nm with ε_max_ = 39 300 M^-1^ cm^-1^ and this is assignable to π–π* transitions of the aromatic systems (either naphthoquinone system, so heterocyclic *N*-donor ligands). The maxima of the intensive absorption bands, located in the region of 460–472 with ε_max_ = 4 000–4 800 M^-1^ cm^-1^, can be most likely associated with the n–π* transitions and they are probably overlapped with the LMCT charge-transfer transitions. Even the spectral measurements performed for the solutions of a higher concentration (at the concentration of 10^−3^ M) did not reveal the positions of the *d*-*d* transitions.

### SQUID magnetometry

**Fig 3:**
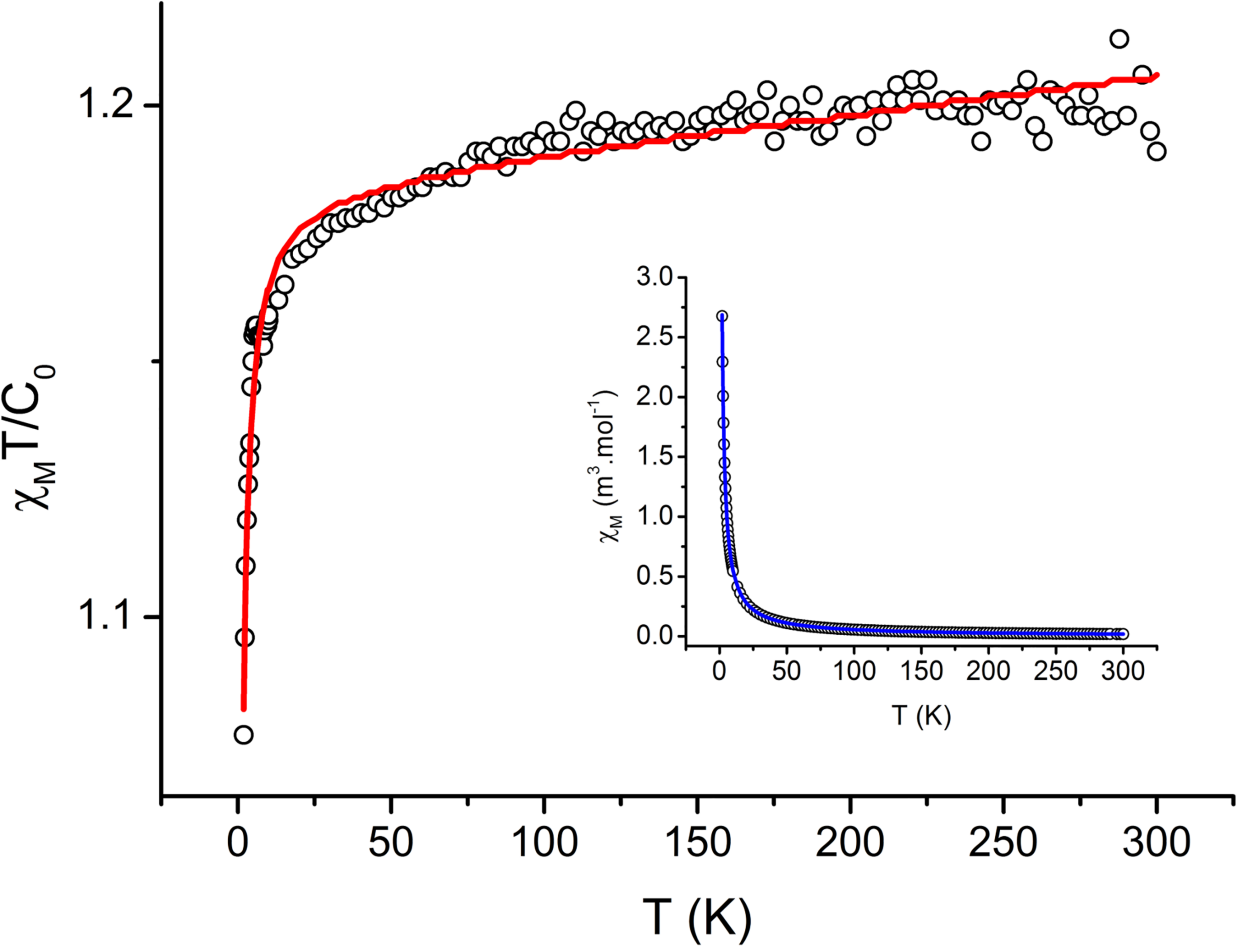
The result of temperature-dependent SQUID magnetometry measurements for complex 5. The curves represent the best fit of experimental data according to the Curie-Weiss law (the best-fit parameters are *g*_*iso*_ = 2.18, *C* = 5.585×10^−6^ m^3^.mol^-1^, and θ **=** -0.181 K with a temperature-independent paramagnetism term a_TIP_ = 3.40×10^−8^ m^3^.mol^-1^). C_0_ = 4.7141997×10^−6^ m^3^.mol^-1^.

The solid-state temperature dependence of magnetic susceptibility was measured over the temperature range of 300–1.9 K for the selected complexes (**1**–**3**, and **5**). As a representative result of the temperature dependence of magnetic susceptibility for complex **5** is shown below (Fig 3).

In all the cases, the complexes behaved as paramagnets and followed the Curie-Weiss law (for the best fit parameters, see Table 2) in the wide range of temperatures. All the complexes showed a week antiferromagnetic exchange, probably mediated through the non-covalent interactions stabilizing the crystal structures of the complexes. In the case of aqua-complex, the participation of hydrogen bonds in the more intense antiferromagnetic exchange was evident at low temperature (1.9–3.0 K).

**Table 2:** The result of the analysis of SQUID magnetometry results by fitting to the Curie-Weiss law.

Complex	*g*_*iso*_	*C* [×10^−6^ m^3^.mol^-1^]	θ [K]	a_TIP_ [×10^−9^ m^3^.mol^-1^]	Fit error (%)
**1**	2.19	5.634	-0.464	0.222	0.71
**2**	2.17	5.517	-0.175	0.087	0.63
**3**	2.20	5.694	-0.366	-0.396	0.48
**5**	2.18	5.585	-0.181	0.340	0.39
Aqua-complex	2.18	5.609	-0.706	0.532	3.92

### X-ray structure of complex 2

**Fig 4:**
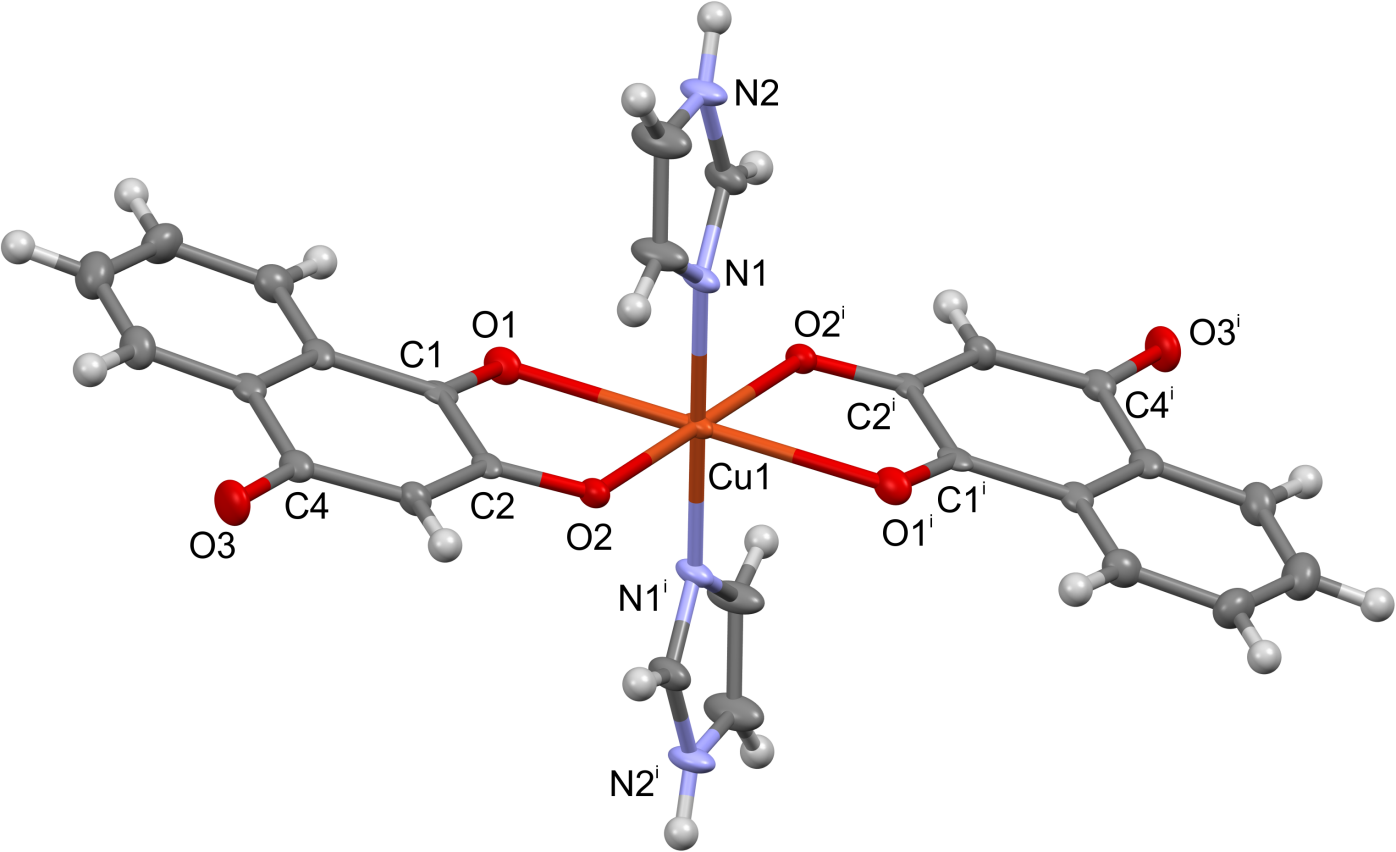
The molecular structure of complex 2. Symmetry code used: ^i^ = -x+1,-y,-z+1.

The molecular structure of complex **2**, [Cu(Law)_2_(Im)_2_], is shown in Fig 4. The molecule of the complex is centrosymmetric with the Cu(II) atom lying on the center of inversion. The central atom of Cu(II) adopts a distorted octahedral geometry with the O_4_N_2_ donor set and is coordinated by two quinonato ligands in the basal plane and by two imidazole ligands in the apical positions. The two of the Cu–O distances (Cu1–O1 = 2.457(3) Å) are significantly longer that those of Cu1–O2 ones (1.971(2) Å). The sum of the van der Waals radii for these atoms is 2.92 Å [25]. Selected bond lengths and angles of complex **2** are given in Table 3, and they are compared with similar complexes involving the Law ligands, i.e. [Cu(Law)_2_(*N*-MeIm)_2_] ([26], CSD Ref. Code TICXIO) and [Cu(Law)_2_(H_2_O)_2_] ([13], CSD Ref. Code FAHNAE).

**Table 3:** Selected bond lengths and angles (Å, °) for 2 and two reference complexes selected from CSD.

Parameter	2	TICXIO^a^	FAHNAE^b^
Cu1–O1	2.457(3)	2.460	2.328
Cu1–O2	1.971(2)	1.964(2)	1.952
Cu1–N1	1.979(3)	2.012(3)	-
Cu1–O4	-	-	1.995
C1–O1	1.227(4)	1.212(5)	1.226(4)
C2–O2	1.285(4)	1.290(4)	1.297(4)
C4–O3	1.254(4)	1.228(7)	1.235(5)
O1–Cu1–O2	74.37(9)	74.57	77.05
O2–Cu1–N1	82.18(7)	89.56(12)	89.13

^a^ The data obtained from CSD for the complex [Cu(Law)_2_(*N*-MeIm)_2_] ([26], CSD Ref. Code TICXIO)

^b^ The data obtained from CSD for the complex [Cu(Law)_2_(H_2_O)_2_] ([13], CSD Ref. Code FAHNAE).

The crystal structure of complex **2** is stabilized by a network of the hydrogen bonds N2–H2a^…^O3^i^, N2^ii^–H2a^ii…^O3^iii^, N2^iv^–H2a^iv…^O3, and N2^v^–H2a^v…^O3^ii^, symmetry codes: (i) 1/2-x,1/2-y,1/2+z; (ii) 1–x,-y,1-z; (iii) 1/2+x,1/2+y,1/2-z; (iv) 1/2-x,1/2-y,z-1/2; (v) 1/2+x,y-1/2,3/2-z (see S11 Fig and S1 Table), connecting the individual molecules into a 2D layers. The structure is further stabilized by the non-covalent C–H^…^O interactions C12–H12a^…^O1^vi^, and C7–H7a^…^O2^vii^, symmetry codes: (vi) x-1/2,1/2-y,1-z; (vii) x,1+y,z (see S12 Fig), forming a 3D supramolecular structure.

### *In vitro *cytotoxicity

The *in vitro* cytotoxicity of complexes **1**–**7** was evaluated as a first step preceding the further *in vitro* analyses of anti-inflammatory activity. Due to low solubility of the complexes in the cultivation medium, we dissolved the complexes either in DMF or PBS to produce the master solution, which was successively diluted by culture medium to produce the final concentrations of the complex for cytotoxicity testing. The maximum content of the solvent in the final solutions was lower than 0.1% (v/v). All the complexes showed cytotoxic effect on THP-1 cell line with the IC_50_ values higher than 10 **μ**M. Lawsone alone demonstrated only negligible cytotoxic effect at 10 **μ**M concentration as the viability of the THP-1 cells was lowered to ca. 80% (see the dose-viability curves, S1 Fig).

### Anti-inflammatory activity *in vitro*

#### Effect of complexes on NF-*κ*B activation

Lawsone is one of the main secondary metabolites isolated from the henna tree (*Lawsonia inermis* L., Lythraceae), which is used in folk medicine for treatment of inflammatory diseases [27–28]. Moreover, it is a well-known fact, that several copper(II) complexes have shown anti-inflammatory potential and ability to act by different mechanism of action [29–30]. In this study, we decided to evaluate the anti-inflammatory potential of mixed-ligand Cu(II) complexes involving lawsone and selected *N*-donor ligands, chosen from azine and azole groups of heterocyclic compounds, both by *in vitro* and *in vivo* methods.

**Fig 5:**
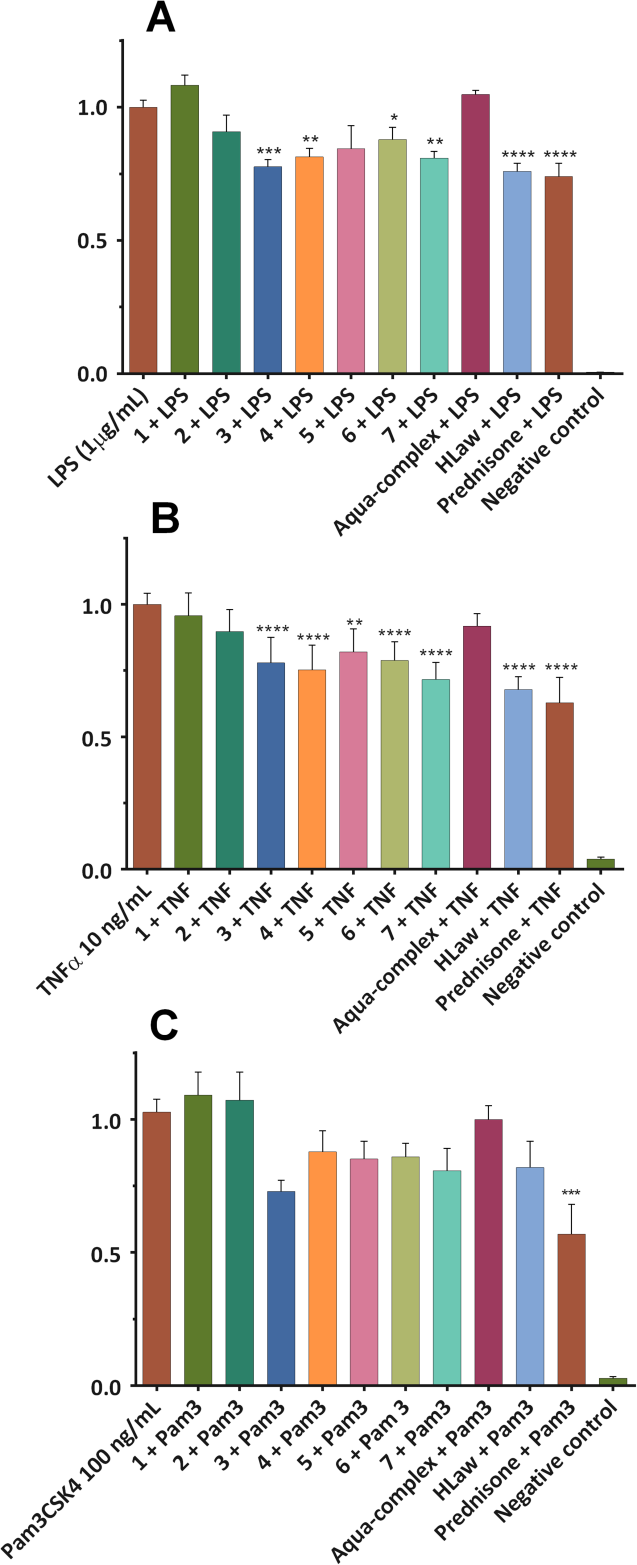
Effect of complexes 1–7 on the NF-*κ*B activity. THP1-XBlue™-MD2-CD14 cell line were pre-treated with the complexes (100 nM, dissolved in PBS), [Cu(Law)_2_(H_2_O)_2_]·0.5H_2_O, HLaw and prednisone (1 μM, dissolved in DMSO) for 1 h. Subsequently, LPS (1 **μ**g/mL) (A) or TNF-α (10 ng/mL) (B) or Pam3CSK4 (100 ng/mL) (C) was added [except for the *control* cells (basal)] to trigger the activation of NF-*κ*B. After 24 h, the activity of NF-*κ*B was evaluated based on the amount of the secreted alkaline phosphatase measured spectrophotometrically. The results are expressed as the mean ± S.E.M. for six independent experiments. ** Indicates a significant difference in comparison with the vehicle-treated cells *p* < 0.01, and **** indicates a significant difference in comparison with the vehicle-treated cells *p* < 0.0001.

The *in vitro* anti-inflammatory potential of the complexes was evaluated by their ability to modulate the activity of one of the key pro-inflammatory transcription factors, the nuclear factor-*κ*B (NF-*κ*B) after induction by three different activators (TNF-α, LPS, and Pam3CSK4, respectively), thus modelling the different signaling pathways of NF-*κ*B activation (see Fig 5).

The first activator was bacterial lipopolysaccharide (LPS, 1 **μ**g/mL), a Toll-like receptor-4 (TLR-4) agonist, which simulates the immune response of human body against the bacteria [31]. Complex **1** did not affect the activity of NF-*κ*B stimulated by LPS, complexes **2** and **6** non-significantly decreased its activity by 10%, and 12%, respectively. All the remaining complexes (**3**, **4**, and **7** applied at the 100 nM concentration), including lawsone (applied at the 100 nM concentration) were able to significantly attenuate the NF-*κ*B activation by 19–24% (Fig 5A). In comparison, the synthetic corticoid steroid prednisone, clinically used for the treatment of inflammatory diseases (applied at the 1 **μ**M concentration), significantly decreased the activity of NF-*κ*B by 26%.

The activation of NF-*κ*B by the second activator, a cytokine tumor necrosis factor alpha (TNF-α), represents an aseptic inflammatory response model, proceeds *via* the activation of the appropriate receptor (TNFR). Complexes **3**–**7** were able to significantly reduce the activity of NF-*κ*B by 18-28%. In this case, however, the pure lawsone reached similar effect as prednisone as these two compounds decreased the NF-*κ*B by 32%, and 37%, respectively (Fig 5B).

The third pathway of the NF-*κ*B activation was induced by the synthetic triacylated lipoprotein Pam3CSK4. Recognition of Pam3CSK4, which mimics the cell wall components found in both Gram positive and Gram negative bacteria, is mediated by TLR2 which cooperates with TLR1 through their cytoplasmic domain to induce the signaling cascade leading to the activation of NF-*κ*B [32–33]. The effect of complexes **1**–**7** on the above-mentioned activation pathway was only marginal. The complexes **3**–**7** decreased the activation of NF-*κ*B by 15–29%, while the complex **3** was the most efficient (Fig 5C). Also in this case, similar to other routes of the NF-*κ*B activation, the complexes **1** and **2** showed no noticeable effect.

#### Effect of complexes on the induction of intracellular oxidative stress

**Fig 6:**
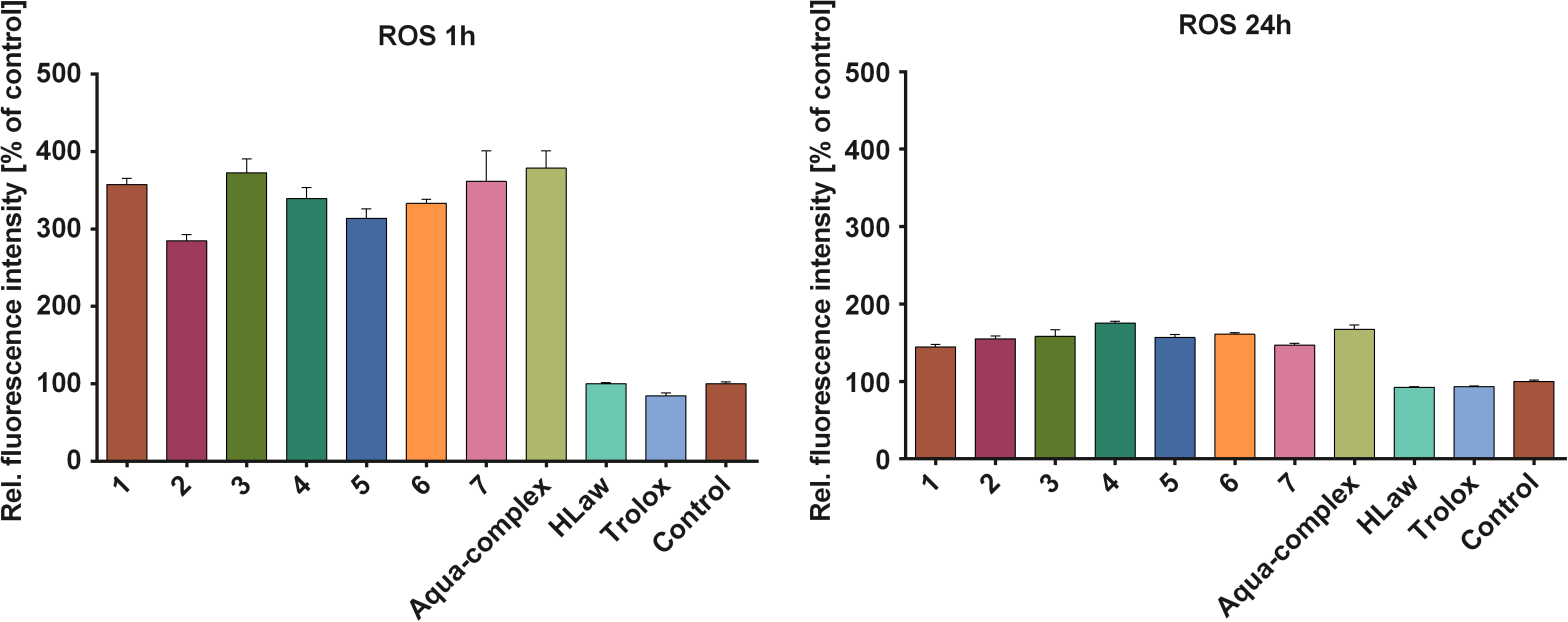
The pro-oxidative effect of complexes 1–7, reference aqua-complex and lawsone determined by dichlorofluorescein method. THP1-XBlue™-MD2-CD14 cell line were treated with the complexes, or standard antioxidant Trolox® at the final concentration of 100 nM for 1 h (left diagram) and 24 h (right diagram). The data represent the relative increase of fluorescence intensity after the addition of DCFH-DA in comparison with the untreated control. The results are expressed as the mean values ± S.E.M. for three independent experiments.

The ability of complexes 1–7 to induce the intracellular oxidative stress was studied by the dichlorofluorescein method, 1h and 24 h after the addition of the complexes into cell medium. By comparing the relative fluorescence intensities obtained after the addition of DCFH-DA in these two time-frames, it is evident that all the complexes caused the burst of ROS production at the beginning and the production of ROS was nearly diminished 24 h after the addition (see Fig 6). The combination of pro-oxidant action and the anti-inflammatory activity is not very common; however, it has been described previously for some non-steroidal anti-inflammatory drugs and their reactive metabolites [34–35], and specifically for two new anti-inflammatory drugs flunoxaprofen and benoxaprofen [36–37].

#### Effect of complexes on TNF-α secretion

**Fig 7:**
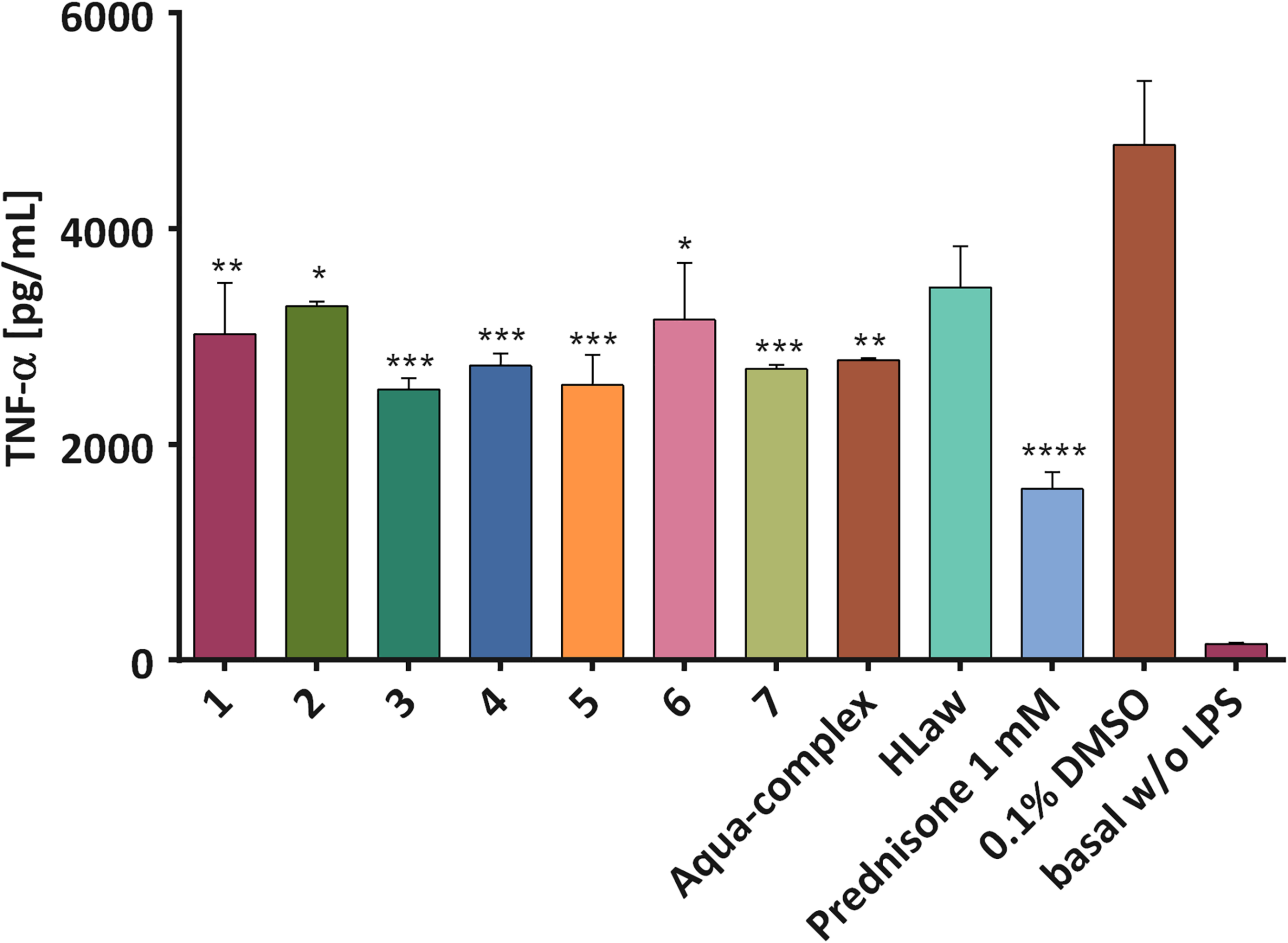
The effect of the tested complexes on the secretion of TNF-α. THP-1 macrophages were pre-treated with the compounds **1**–**7**, [Cu(Law)_2_(H_2_O)_2_]·0.5H_2_O (aqua-complex), HLaw (100 nM, dissolved in PBS) and prednisone (1 **μ**M, dissolved in DMSO) for 1 h. Subsequently, LPS (1 **μ**g/mL) was added [except for the *control* cells (basal)] to trigger the secretion of the pro-inflammatory cytokine TNF-α. After 24 h, the amount of the secreted TNF-α was evaluated by ELISA. The results are expressed as the mean values ± S.E.M. for three independent experiments. * Indicates a significant difference in comparison with the vehicle-treated cells *p* < 0.05, ** indicates a significant difference in comparison with the vehicle-treated cells *p* < 0.01, *** indicates a significant difference in comparison with the vehicle-treated cells *p* < 0.001, and **** indicates a significant difference in comparison with the vehicle-treated cells *p* < 0.0001.

Cytokine TNF-α is a prominent pro-inflammatory signaling peptide, which is under transcription control of NF-*κ*B [38]. All the tested complexes decreased significantly its expression in LPS-stimulated cells by 31–48% (see Fig 7). The greatest potential showed the complex 3. Interestingly, pure lawsone reduced the TNF-α level non-significantly only by 28%.

It is a known fact, that naphthoquinones can attenuate the NF-κB activity and thus reduce the expression of pro-inflammatory genes. [39] On the other hand, they could also involve in the processes leading to the induction of the oxidative stress, for example in the presence of the redox-active transition metals. [40] As a consequence of the oxidation of some intermediates (e.g. IKKβ or p50), involved in the NF-*κ*B activation pathway, the activity of NF-*κ*B can, in fact, be decreased. [41] In the case of the tested complexes, we can observe both effects. Moreover, we can hypothesize that also other molecular mechanisms could influence the processes of TNF-α transcription/translation, or secretion, for example the oxidative stress can impede the processes of exocytosis [42] or proteolytic activation of membrane-bound TNF-α molecules. The tested complexes could also affect TNF-α transcription and/or translation which could explain the discrepancy between NF-*κ*B activation and TNF-α secretion.

From this point of view, the Cu(II)-lawsone complexes represent promising group of new potential anti-inflammatory drugs.

### Anti-inflammatory activity *in vivo*

**Fig 8:**
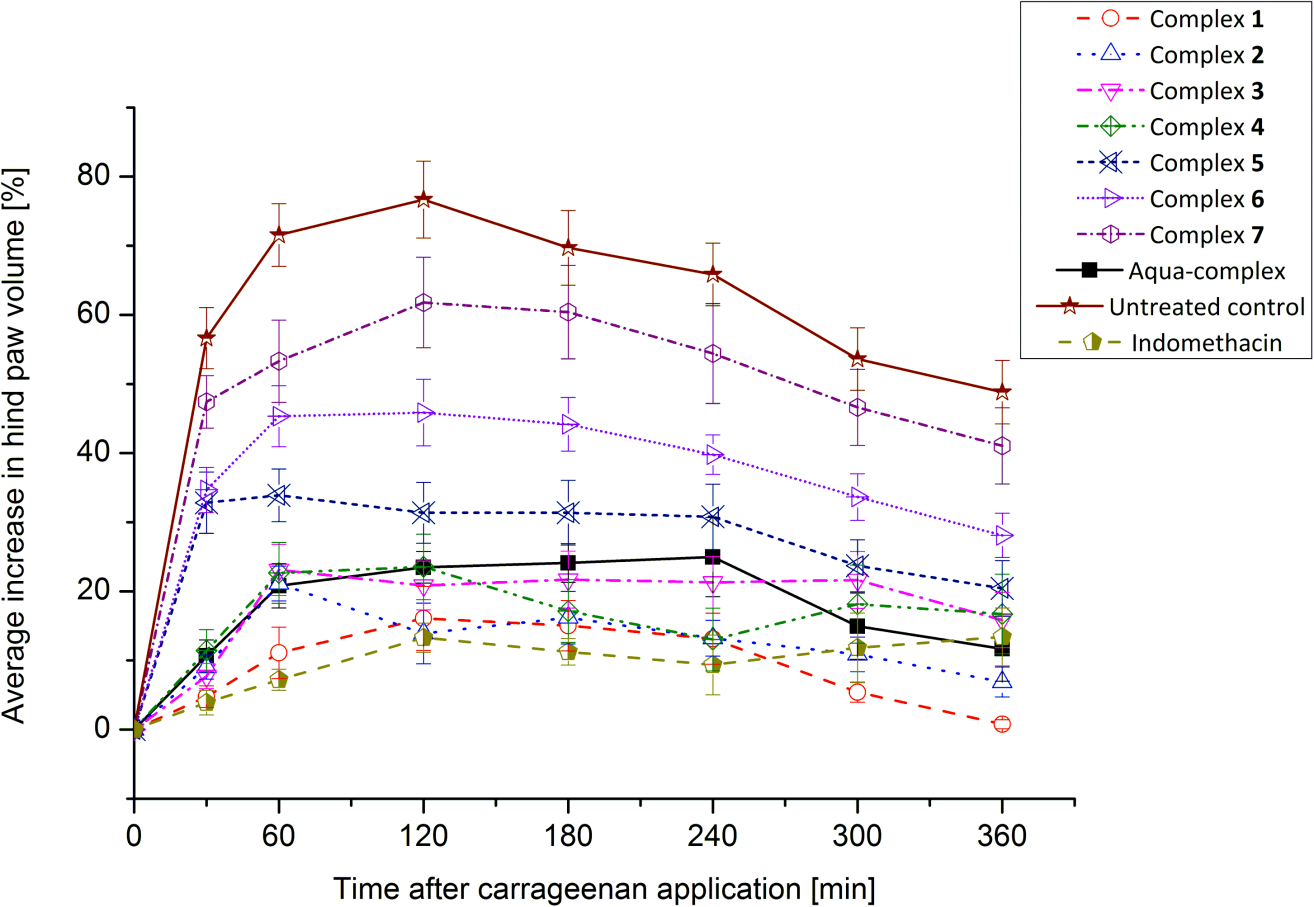
The time-resolved changes in the average volume of the hind paws of rats. The values are expressed as average values ± S.E.M. calculated for 7 animals in each experimental group. The copper(II) complexes were applied *i*.*p*. at the dose corresponding to 40 mmol Cu/kg (ca. 20 mg/kg), and indomethacin at the *i*.*p*. dose of 5 mg/kg.

In continuation of the in vitro testing of anti-inflammatory activities of complexes **1**–**7**, all the copper(II) complexes were included to *in vivo* tests of anti-inflammatory activity using the carrageenan-induced hind paw edema model. The effect of the tested complexes on the acute inflammatory process (leading to swelling of the hind paw), caused by the carrageenan injection, was evaluated plethysmometrically. The clinically used non-steroidal anti-inflammatory drug indomethacin was used as a primary standard for anti-inflammatory activity. The overview of time-resolved antiedematous activity profiles of the tested compounds is summarized in Fig 8.

The results of antiedematous activity showed very similar pharmacological profiles of complexes **1**–**4** and aqua-complex [Cu(Law)_2_(H_2_O)_2_]·0.5H_2_O with the reference drug indomethacin. The most active complex **1** was able to diminish completely the swelling of the hind paw at the end of the experiment (after 6 h). With respect to the structural similarity of all the tested complexes, the similar mechanism of action can be expected, while the *N*-donor ligands (L_N_) might play some additional role in these processes. For example, the 2-aminopyridine [43] and 4-aminopyridine [44] were found to be anti-inflammatory active as metabolites of anti-inflammatory drugs [43] or as a complementary mechanism in the treatment of neurodegenerative diseases. [44] On the other hand, their metabolism relates to the formation of several reactive intermediates and free radicals [45].

**Fig 9:**
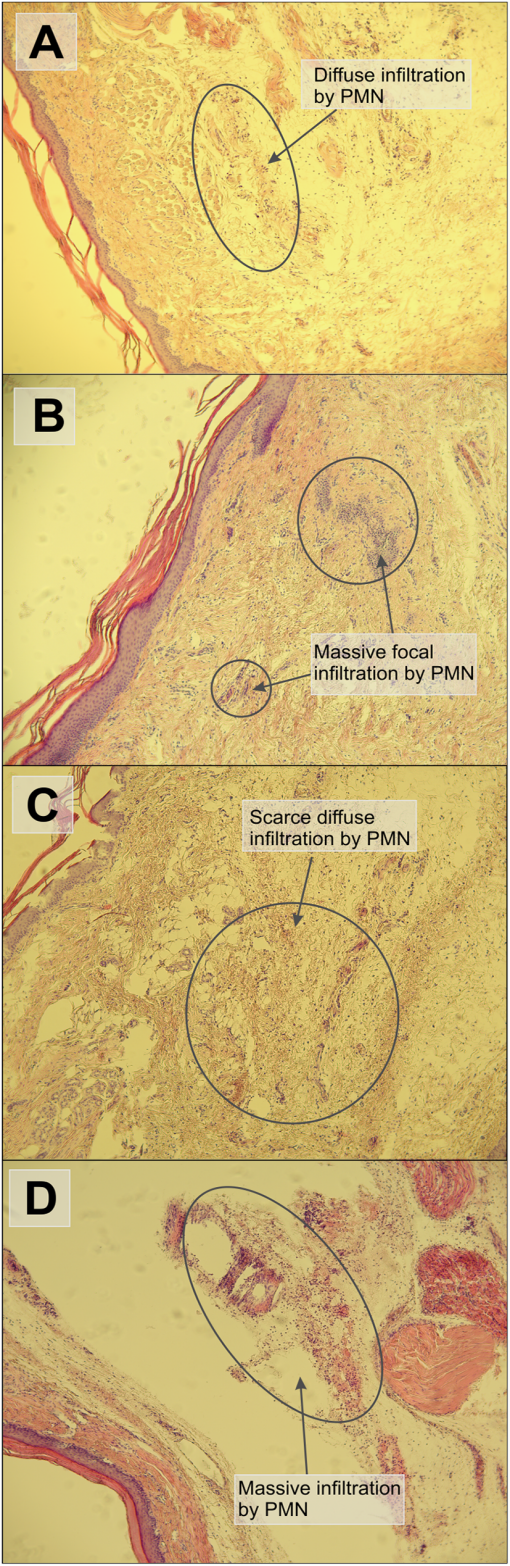
Histological evaluation of inflammatory response in tissue sections of the hind paw, stained by hematoxylin/eosin (40x magnification). The section of plantar tissues from the groups pretreated with complex **1** (A) or indomethacin (C) with the weak inflammatory response in the hypodermis with scarce PMN infiltrate. The tissue sections from the group exposed to complex **7** (B) and 10% DMF solution (control, D) with the strong inflammatory reaction in the hypodermis with massive focal PMN infiltrate.

The results obtained by plethysmometric method were further confirmed by the histopathological analysis of tissue samples isolated from the plantar area of hind paws. The histopathological changes in tissues, stained by the standard hematoxylin/eosin staining for the most active complex **1**, the least active complex **7**, indomethacin and control group (see Fig 9), were evaluated on basis of the the presence of the inflammation infiltrate, which contained mainly neutrophils (polymorphonuclear cells—PMN). These changes provided evidence of the acute inflammation, which were manifested by the massive presence of PMN cells, in the samples from the control group (see Fig 9D) and the group pretreated with complex **7** (see Fig 9B). On the other hand, the PMN infiltration was mainly scarce and diffuse in samples obtained from indomethacin (see Fig 9C) and complex **1** (see Fig 9A) treated groups. Both these substances significantly decreased the inflammatory reaction.

## Conclusions

A series of mixed-ligand copper(II) complexes (**1**–**7**), involving the lawsone and heterocyclic *N*-donor ligands, has been studied for anti-inflammatory activity on *in vitro* and *in vivo* levels. Complexes **3**–**7** showed the ability to suppress significantly the activation of nuclear factor-*κ*B (NF-*κ*B) both by lipopolysaccharide (LPS) and by TNF-α in the similar manner as the reference drug prednisone, even at the 100 nM level. Moreover, all the studied complexes **1**–**7** decreased significantly the levels of the secreted TNF-α after the LPS activation of the THP-1 cells. The complexes also strongly induced the intracellular production of ROS. The *in vivo* testing on hind-paw edema model on rats revealed the significant anti-edematous effect of complexes **1**–**3**, which diminished the formation of edema in the similar way as the reference drug indomethacine. The pharmacological profile of complexes **1**–**3** resembles that of anti-inflammatory drug benoxaprofen, also known to cause intracellular production of ROS. To conclude, we may state that the obtained results clearly enrich the knowledge about the biological activities of copper(II) complexes and thus, they might serve as a clue for the development of new anti-inflammatory active complexes involving the 1,4-naphthoquinone ligands.

## Supporting information

S1 Text Synthesis and characterization of the reference complex [Cu(Law)_2_(H_2_O)_2_]·0.5H_2_O. (PDF)

S1 Fig The dose-viability curves obtained for the copper(II) complexes by *in vitro* cytotoxicity screening against THP-1 cells. The red dashed line represents the viability level of 50%.
(TIF)

S2 Fig The results of simultaneous TG/DSC analysis of complex 7 showing TG and DSC curves and the interpretation of calculated and observed weight losses. (TIF)

S3 Fig The results of simultaneous TG/DSC analysis of the aqua-complex [Cu(Law)_2_(H_2_O)_2_]·0.5H_2_O showing TG and DSC curves and the interpretation of calculated and observed weight losses. (TIF)

S4 Fig The ESI+ mass spectrum of methanol solution of complex 1. (TIF)

S5 Fig The ESI+ mass spectrum of methanol solution of complex 3. (TIF)

S6 Fig Infrared spectrum of complex 1 measured by the ATR technique in the region of 650–4000 cm^-1^. The maxima of the main peaks are noted.
(TIF)

S7 Fig Infrared spectrum of complex 7 measured by the ATR technique in the region of 650–4000 cm^-1^. The maxima of the main peaks are noted.
(TIF)

S8 Fig Diffuse-reflectance spectrum of complex 1 measured by Nujol technique in the range of 400–1000 nm. The remark sh means shoulder.
(PNG)

S9 Fig Diffuse-reflectance spectrum of complex 2 measured by Nujol technique in the range of 400–1000 nm. The remark sh means shoulder.
(PNG)

S10 Fig The comparison of UV-Visible spectra of the complexes measured in methanol solutions at the concentration of 5×10^−5^ M (panel A), and 5×10^−4^ M (panel B). (TIF)

S11 Fig Fragment of the crystal structure of complex 2, showing the network of the hydrogen bonds N–H…O (blue dashed lines) and formation of a 2D layer. A distance *d*(N2…O3^i^) = 2.778(4) Å (symmetry code: (i) 1/2-x,1/2-y,1/2+z).
(TIF)

S12 Fig Fragment of the crystal structure of complex 2, showing the non-covalent intermolecular C12–H12a…O1^vi^ and C7–H7a…O2^vii^ contacts (blue dashed lines) and a part of a 3D supramolecular structure. The distances of *d*(C12…O1^i^) = 3.199(5) Å and *d*(C7…O2^ii^) = 3.256(5) Å (symmetry codes: (vi) x-1/2,1/2-y,1-z; (vii) x,1+y,z).
(TIF)

S1 Table The interatomic parameters (in Å and °) of selected non-covalent interactions in the crystal structure of complex 2. (PDF)

## References

[1] Pradhan R, Dandawate P, Vyas A, Padhye S, Biersack B, Schobert R, et al From Body Art to Anticancer Activities: Perspectives on Medicinal Properties of Henna, Curr. Drug Targets 2012; 13: 1777–1798. 23140289 10.2174/138945012804545588

[2] Singh DK, Luqman S, Mathur AK. Lawsonia inermis L.–A commercially important primaeval dying and medicinal plant with diverse pharmacological activity: A review. Ind. Crop. Prod. 2015; 65: 269–286.

[3] Biradar S, Veeresh B. Protective effect of lawsone on L-Arginine induced acute pancreatitis in rats. Indian J. Exp. Biol. 2013; 51: 256–261. 23678547

[4] Ali BH, Bashir AK, Tanira MOM. Anti-inflammatory, antipyretic, and analgesic effects of Lawsonia inermis L (Henna) in rats. Pharmacology 1995; 51: 356–363. 8966192 10.1159/000139347

[5] Marzin D, Kirkland D. 2-Hydroxy-1,4-naphthoquinone, the natural dye of Henna, is non-genotoxic in the mouse bone marrow micronucleus test and does not produce oxidative DNA damage in Chinese hamster ovary cells. Mutat. Res. 2004; 560: 41–47. doi: 10.1016/j.mrgentox.2004.02.004 15099823 10.1016/j.mrgentox.2004.02.004

[6] Salunke-Gawali S, Rane S, Boukheddaden K, Codjovi E, Linares J, Varret F, et al Thermal, magnetic and electrochemical properties of polymeric copper complexes of 2-hydroxy-1,4-naphthoquinone and its methyl derivative. Ind. J. Chem. 2004; 43A: 2563–2567.

[7] Tabrizi L, Talaie F, Chiniforoshan H. Copper(II), cobalt(II) and nickel(II) complexes of lapachol: synthesis, DNA interaction, and cytotoxicity. J. Biomol. Struct. Dyn. 2017 *Accepted paper* doi: doi: 10.1080/07391102.2016.1254118 27897079 10.1080/07391102.2016.1254118

[8] Valle-Bourrouet G, Ugalde-Saldívar VM, Gómez M, Ortiz-Frade LA, González I, Frontana C. Magnetic interactions as a stabilizing factor of semiquinone species of lawsone by metal complexation. Electrochim. Acta 2010; 55: 9042–9050.

[9] Brandelli A, Bizani D, Martinelli M, Stefani V, Gerbase AE. Antimicrobial activity of 1,4-naphthoquinones by metal complexation. Brazil. J. Pharm. Sci. 2004; 40: 247–253.

[10] Oramas-Royo S, Torrejón C, Cuadrado I, Hernández-Molina R, Hortelano S, Estévez-Braun A, et al Synthesis and cytotoxic activity of metallic complexes of lawsone. Bioorg. Med. Chem. 2013; 21: 2471–2477. doi: 10.1016/j.bmc.2013.03.002 23545136 10.1016/j.bmc.2013.03.002

[11] Tabrizi L, Fooladivanda M, Chiniforoshan H. Copper(II), cobalt(II) and nickel(II) complexes of juglone: synthesis, structure, DNA interaction and enhanced cytotoxicity. Biometals 2016; 29: 981–993. doi: 10.1007/s10534-016-9970-0 27613275 10.1007/s10534-016-9970-0

[12] Babula P, Vanco J, Krejcova L, Hynek D, Sochor J, Adam V, et al Voltammetric Characterization of Lawsone-Copper(II) Ternary Complexes and Their Interactions with dsDNA. Int. J. Electrochem. Sci. 2012; 7: 7349–7366.

[13] Salunke-Gawali S, Rane SY, Puranik VG, Guyard-Duhayon C, Varret F. Three dimensional hydrogen-bonding network in a copper complex of 2-hydroxy-1,4-naphthoquinone: structural, spectroscopic and magnetic properties. Polyhedron 2004; 23: 2541–2547.

[14] Bruker. Apex3. Bruker AXS Inc., Madison, Wisconsin, USA, 2015.

[15] Sheldrick GM. Crystal structure refinement with SHELXL. Acta Crystallogr. C 2015; 71: 3–8. 10.1107/S2053229614024218 PMC4294323 25567568

[16] Macrae CF, Bruno IJ, Chisholm JA, Edgington PR, McCabe P, Pidcock E, et al Mercury CSD 2.0—New features for the visualisation and investigation of crystal structures. J. Appl. Crystallogr. 2008; 41: 466–470.

[17] Vanco J, Galikova J, Hosek J, Dvorak Z, Parakova L, Travnicek Z. Gold(I) Complexes of 9-Deazahypoxanthine as Selective Antitumor and Anti-Inflammatory Agents. Plos One 2014; 9: e109901 doi: 10.1371/journal.pone.0109901 25333949 10.1371/journal.pone.0109901 PMC4198181

[18] Wang H, Joseph JA. Quantifying Cellular Oxidative Stress by Dichlorofluorescein Assay using Microplate Reader. Free Rad. Biol. Med. 1999; 27: 612–616. 10490282 10.1016/s0891-5849(99)00107-0

[19] Kalyanaraman B, Darley-Usmar V, Davies KJA, Dennery PA, Forman HJ, Grisham MB, et al Measuring reactive oxygen and nitrogen species with fluorescent probes: challenges and limitations. Free Rad. Biol. Med. 2012; 52: 1–6. doi: 10.1016/j.freeradbiomed.2011.09.030 22027063 10.1016/j.freeradbiomed.2011.09.030 PMC3911769

[20] Garber JC, Barbee RW, Bielitzki JT, Clayton LA, Donovan JC, et al Guide for the Care and Use of Laboratory Animals, 8th ed., Washington: The National Academies Press, USA, 2011, 246 p.

[21] Zimmermann M. Ethical guidelines for investigations of experimental pain in conscious animals. Pain 1983; 16: 109–110. 6877845 10.1016/0304-3959(83)90201-4

[22] Chang HZ, Sheu MJ, Yang CH, Leu ZC, Chang YS, Peng WH, et al Analgesic effects and the mechanisms of anti-inflammation of hispolon in mice. Evid. Based Complement. Alternat. Med. 2011; Article ID 478246. 10.1093/ecam/nep027 PMC3136186 19349477

[23] Pouchert ChJ. The Aldrich Library of Infrared Spectra, 3^rd^ ed., Aldrich Chemical Company, Milwaukee, USA, 1981, 1873 p.

[24] Lever ABP. Inorganic Electronic Spectroscopy, 2nd ed., Elsevier Publishing Co., Amsterdam, 1984.

[25] Bondi A. van der Waals Volumes and Radii. J. Phys. Rev. 1964; 68: 441–451.

[26] Casanova I, Sousa-Pedrares A, Viqueira J, Duran ML, Romero J, Sousa A, et al Electrochemical synthesis and structural characterization of homoleptic and heteroleptic cobalt, nickel, copper, zinc and cadmium compounds with the 2-hydroxy-1,4-naphthoquinone ligand. New J. Chem. 2013; 37: 2303–2316.

[27] Singh S, Srivastava NM, Modi NT, Saifi AQ. Anti-inflammatory activity of Lawsonia inermis. Curr. Sci. 1982; 51: 470–471.

[28] Semwal RB, Semwal DK, Combrinck S, Cartwright-Jones C, Viljoen A. Lawsonia inermis L. (henna): Ethnobotanical, phytochemical and pharmacological aspects. J. Ethnopharmacol. 2014; 155: 80–103. doi: 10.1016/j.jep.2014.05.042 24886774 10.1016/j.jep.2014.05.042

[29] Duncan C, White AR. Copper complexes as therapeutic agents. Metallomics 2012; 4: 127–138. doi: 10.1039/c2mt00174h 22187112 10.1039/c2mt00174h

[30] Medici S, Peana M, Nurchi VM, Lachowicz JI, Crisponi G, Zoroddu MA. Noble metals in medicine: latest advances. Coord. Chem. Rev. 284 (2015) 329–350.

[31] Guha M, Mackman N. LPS induction of gene expression in human monocytes. Cell Signal. 2001; 13: 85–94. 11257452 10.1016/s0898-6568(00)00149-2

[32] Aliprantis AO, Yang RB, Mark MR, Suggett S, Devaux B, Radolf JD, et al Cell activation and apoptosis by bacterial lipoproteins through toll-like receptor-2. Science 1999; 285: 736–739. 10426996 10.1126/science.285.5428.736

[33] Ozinsky A, Underhill DM, Fontenot JD, Hajjar AM, Smith KD, Wilson CB, et al The repertoire for pattern recognition of pathogens by the innate immune system is defined by cooperation between toll-like receptors. PNAS 2000; 97: 13766–13771. doi: 10.1073/pnas.250476497 11095740 10.1073/pnas.250476497 PMC17650

[34] Galati G, Tafazoli S, Sabzevari O, Chan TS, O'Brien PJ. Idiosyncratic NSAID drug induced oxidative stress. Chem. Biol. Interact. 2002; 142: 25–41. 12399153 10.1016/s0009-2797(02)00052-2

[35] Miyamoto G, Zahid N, Uetrecht JP. Oxidation of diclofenac to reactive intermediates by neutrophils, myeloperoxidase, and hypochlorous acid. Chem. Res. Toxicol. 1997; 10: 414–419. doi: 10.1021/tx960190k 9114978 10.1021/tx960190k

[36] Van Rensburg AJ, Theron AJ, Anderson R. Comparison of the pro-oxidative interactions of flunoxaprofen and benoxaprofen with human polymorphonuclear leucocytes in vitro. Agents Actions 1991; 33: 292–299. 1950816 10.1007/BF01986576

[37] Lukey PT, Anderson R, Dippenaar UH, Benoxaprofen activates membrane-associated oxidative metabolism in human polymorphonuclear leucocytes by apparent modulation of protein kinase C. Br. J. Pharmacol. 1988; 93: 289–294. 2833969 10.1111/j.1476-5381.1988.tb11433.x PMC1853819

[38] Zelova H, Hosek J. TNF-α signalling and inflammation: Interactions between old acquaintances. Inflamm. Res. 2013; 62: 641–651. doi: 10.1007/s00011-013-0633-0 23685857 10.1007/s00011-013-0633-0

[39] Kobayashi K, Nishiumi S, Nishida M, Hirai M, Azuma T, Yoshida H, et al Effects of quinone derivatives, such as 1,4-naphthoquinone, on DNA polymerase inhibition and anti-inflammatory action. Med. Chem. 2011; 7: 37–44. 21235518 10.2174/157340611794072742

[40] McKallip RJ, Lombard C, Sun JP, Ramakrishnan R. Toxicology and applied pharmacology 2010; 247: 41–52. doi: 10.1016/j.taap.2010.05.013 20576514 10.1016/j.taap.2010.05.013

[41] Morgan MJ, Liu ZG. Crosstalk of reactive oxygen species and NF-κB signaling. Cell Res. 2011; 21: 103–115. doi: 10.1038/cr.2010.178 21187859 10.1038/cr.2010.178 PMC3193400

[42] Stanley AC, Lacy P. Pathways for Cytokine Secretion. Physiology 2010; 25: 218–229. doi: 10.1152/physiol.00017.2010 20699468 10.1152/physiol.00017.2010

[43] Hawley RC, Labadie SS, Sjogren EB, Talamas FX. Aminopyrimidine and Aminopyridine Anti-Inflammation Agents. US Patent No. US 6,846,828 B2, 2005.

[44] Franciosi S, Ryu JK, Choi HB, Radov L, Kim SU, McLarnon JG. Broad-Spectrum Effects of 4-Aminopyridine to Modulate Amyloid *β*_1–42_-Induced Cell Signaling and Functional Responses in Human Microglia. J. Neurosci. 2006; 26: 11652–11664. doi: 10.1523/JNEUROSCI.2490-06.2006 17093087 10.1523/JNEUROSCI.2490-06.2006 PMC6674791

[45] Uetrecht JP. Myeloperoxidase as a generator of drug free radicals. Biochem. Soc. Symp. 1995; 61: 163–170. 8660393 10.1042/bss0610163

