# Supplementary material for: In vitro and in vivo anti-inflammatory active copper(II)-lawsone complexes
Source: PLoS One. 2017 Jul 25;12(7):e0181822. doi: 10.1371/journal.pone.0181822 (PMC5526570; doi:10.1371/journal.pone.0181822)
Supplement: S1 Text — (PDF) [file pone.0181822.s001.pdf]

Synthesis and characterization of the reference complex $[Cu(Law)_2(H_2O)_2]\cdot0.5H_2O$

The reference complex was prepared by the modification of published method of S. Salunke-Gawali *et al.*, in our case using a copper(II) acetate monohydrate as a starting material without a need to neutralize the resulting solution with sodium carbonate as originally used by S. Salunke-Gawali *et al* [13]. The crystalline product formed rapidly during the 1 hour of heating to 60°C while stirring in the 65% yield. The crystals of the compound were washed with ethanol (2 x 5 mL) and stored in desiccator over KOH for 2 days. The purity of the complex was checked by elemental analysis and characterized by infrared spectroscopy. The complex with the composition $[Cu(Law)_2(H_2O)_2]\cdot0.5H_2O$ was used as a reference for the further evaluation of complexes **1**–**7**.

$[Cu(Law)_2(H_2O)_2]\cdot0.5H_2O$: Yield: 65%, Anal. calc for $C_{20}H_{15}O_{8.5}Cu$ (Mr= 454.89): C, 52.81; H, 3.32. Found: C, 53.10; H, 3.53. ESI+MS (methanol, *m/z*): 197.0 (calc 197.0) $[Law+Na]^+$, 409.8 (calc. 409.9) $[Cu(Law)_2+H]^+$, 431.8 (calc. 431.9) $[Cu(Law)_2+Na]^+$, 447.8 (calc. 447.9) $[Cu(Law)_2+K]^+$, 644.6 (calc. 644.9) $[Cu_2(Law)_3]^+$, 840.5 (calc. 840.9) $[Cu_2(Law)_4+Na]^+$. IR (ν_{ATR}/cm^{-1}): 3339m, 3033m, 1655m, 1625m, 1583s, 1561s, 1367m, 1336m, 1267s, 1250sh, 1214m, 1120m, 985s, 876m, 840m, 781m, 728s, 665m. UV-Vis (Nujol): λ_{max} (nm) 473sh, 727. UV-Vis (methanol): λ_{max} (nm) ($\varepsilon_{max} \times 10^3$ M^{-1} cm^{-1}) 280 (25.91), 460 (4.16).
