# Supplementary figures and images for: In vitro and in vivo anti-inflammatory active copper(II)-lawsone complexes
Source: PLoS One. 2017 Jul 25;12(7):e0181822. doi: 10.1371/journal.pone.0181822 (PMC5526570; doi:10.1371/journal.pone.0181822)

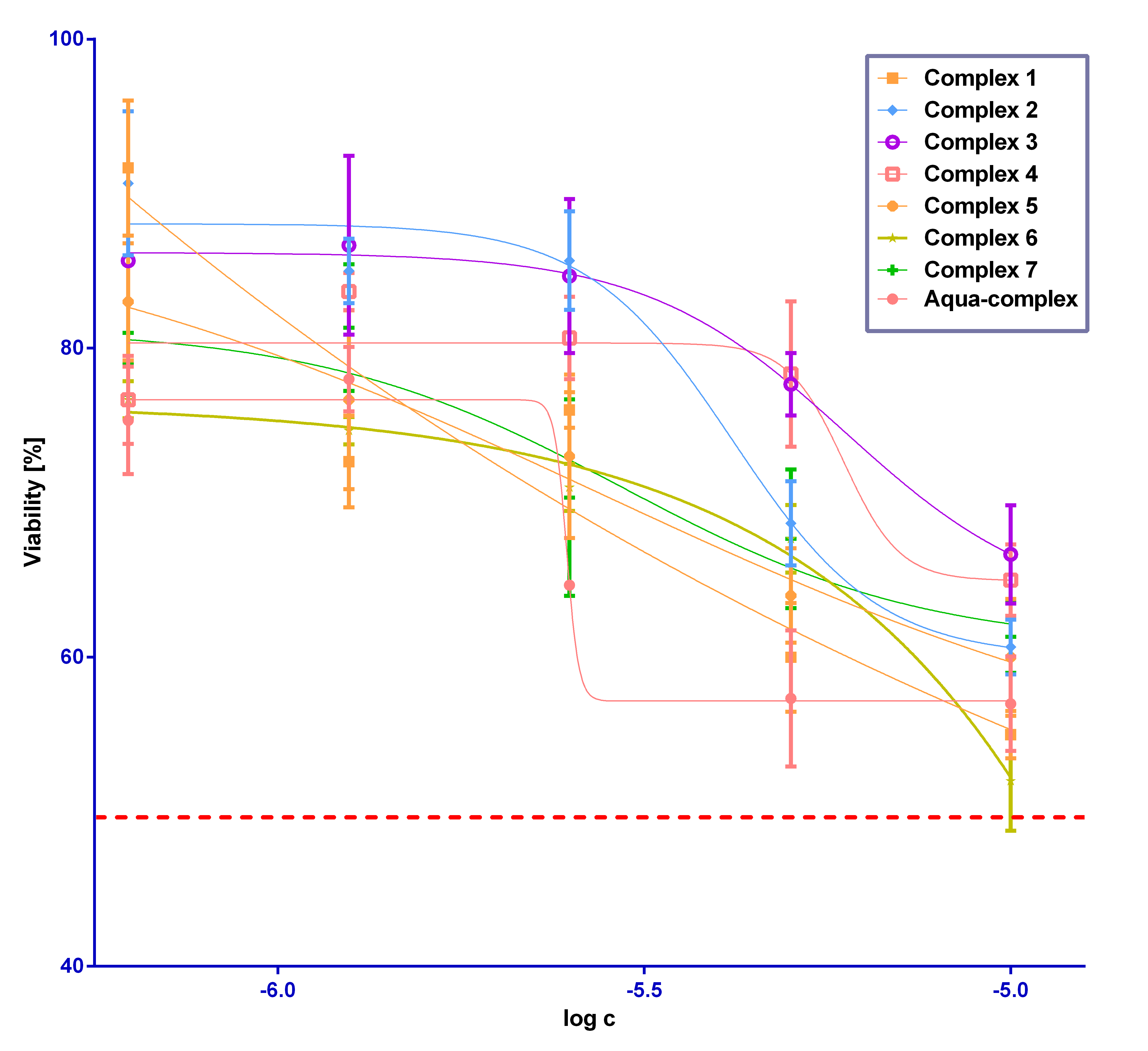

Supplement: S1 Fig — The red dashed line represents the viability level of 50%. (TIF) [file pone.0181822.s002.tif]

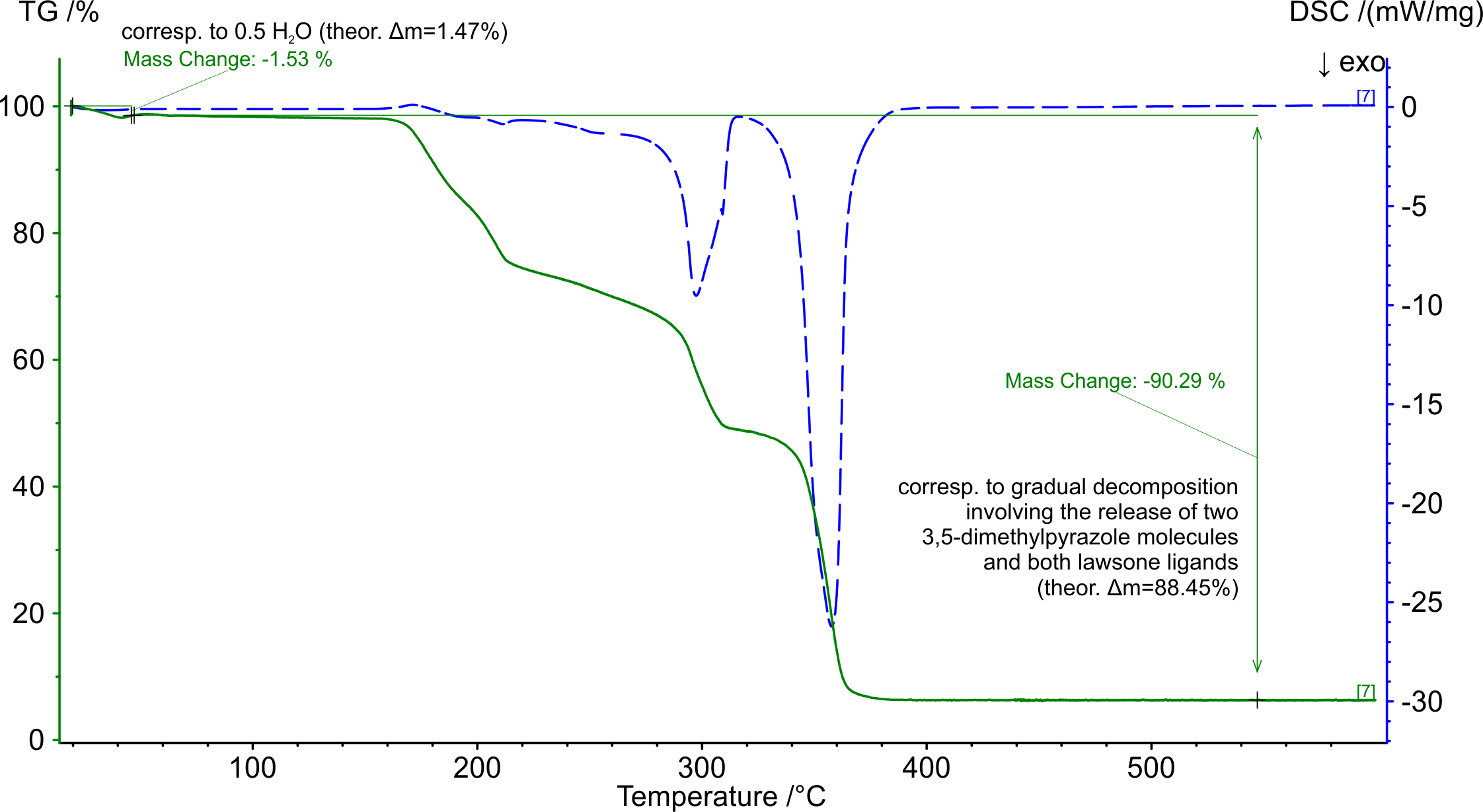

Supplement: S2 Fig — (TIF) [file pone.0181822.s003.tif]

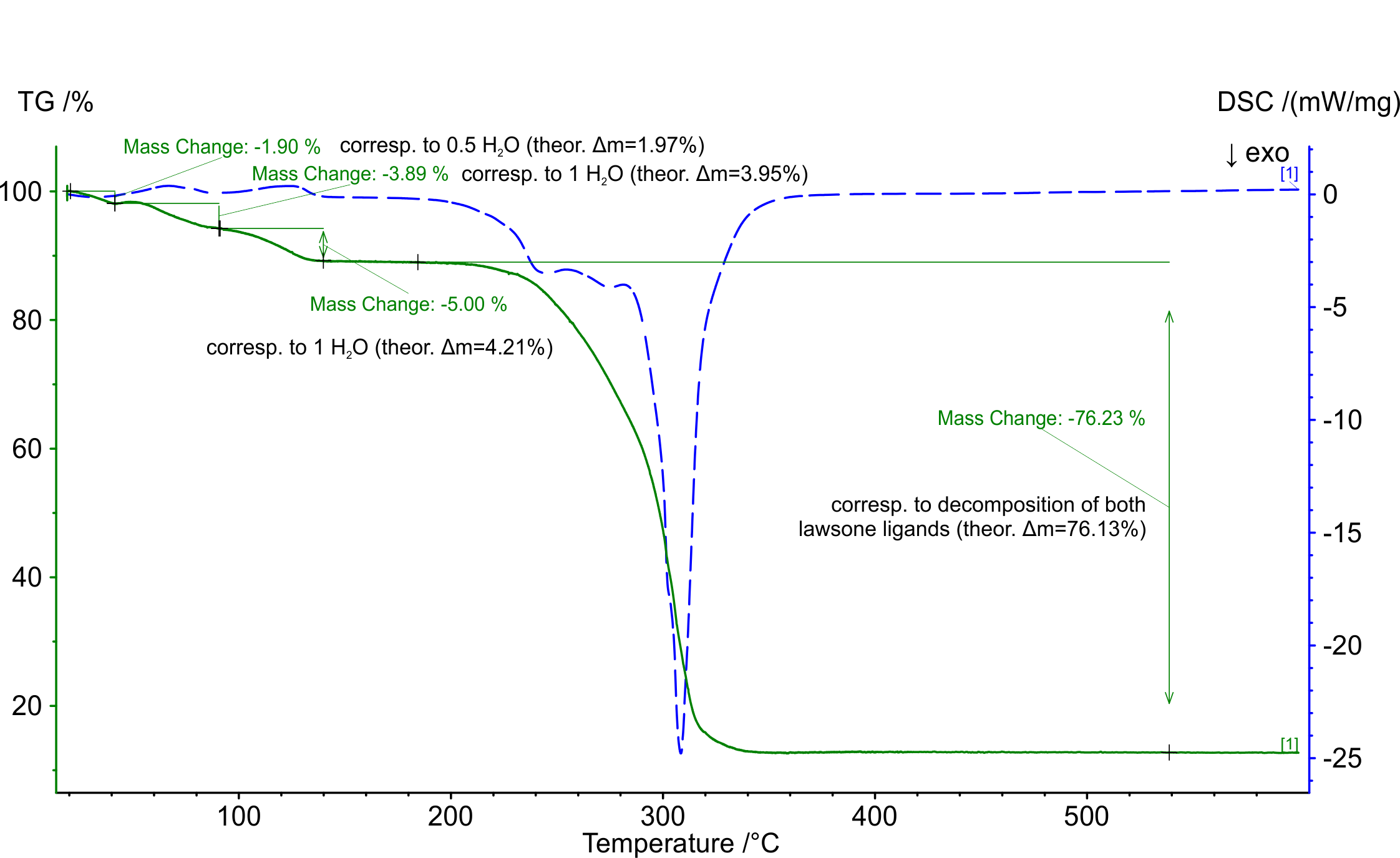

Supplement: S3 Fig — (TIF) [file pone.0181822.s004.tif]

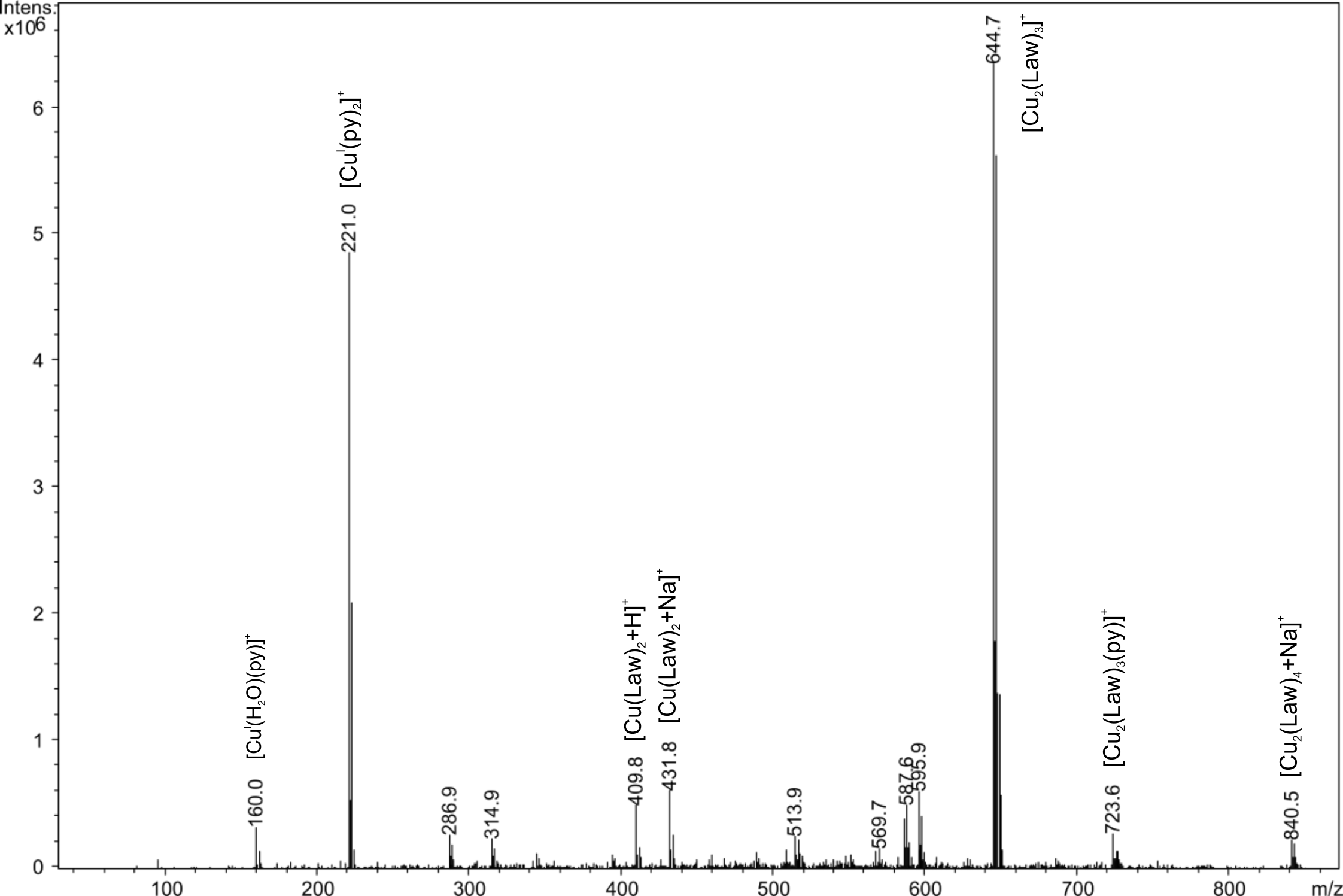

Supplement: S4 Fig — (TIF) [file pone.0181822.s005.tif]

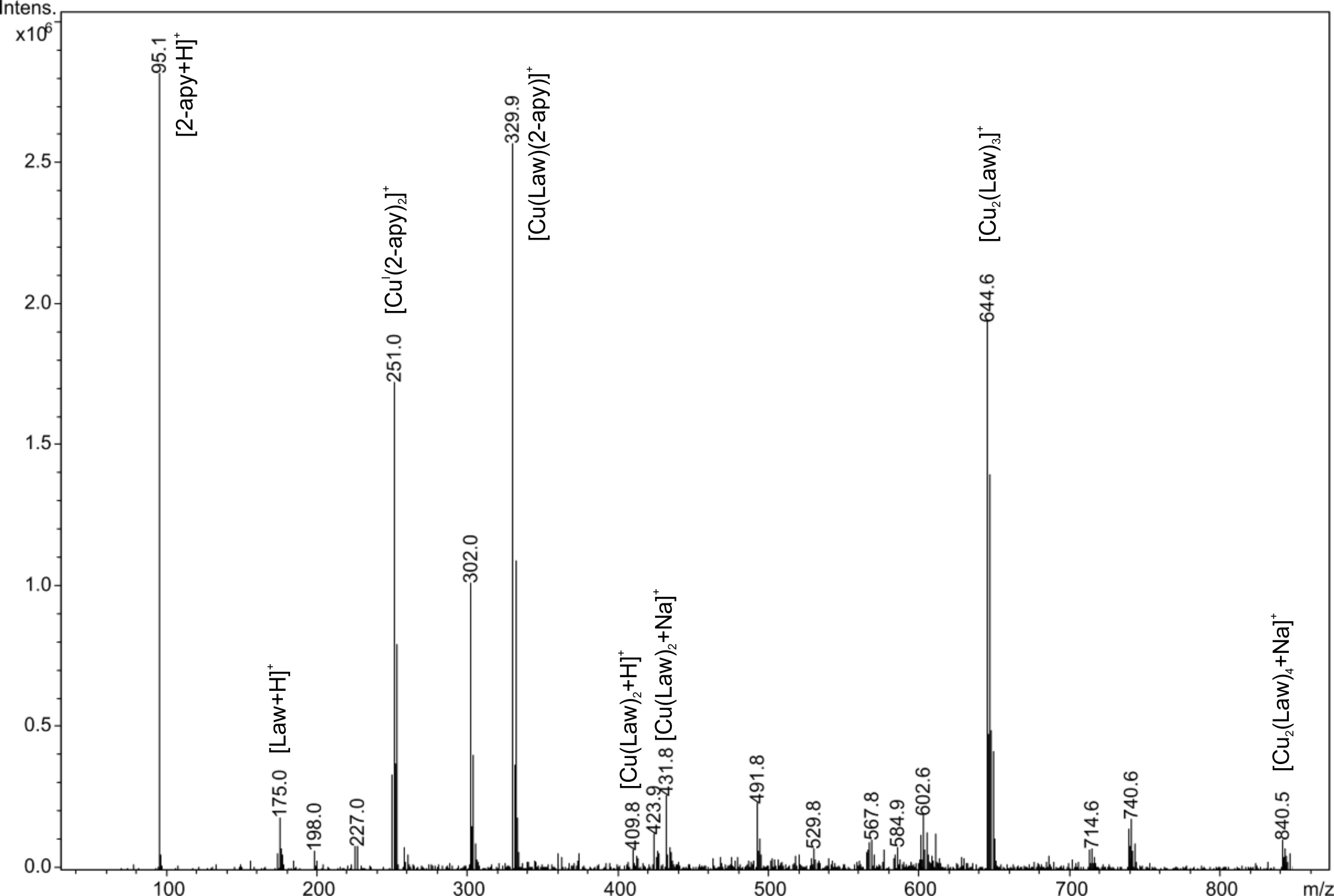

Supplement: S5 Fig — (TIF) [file pone.0181822.s006.tif]

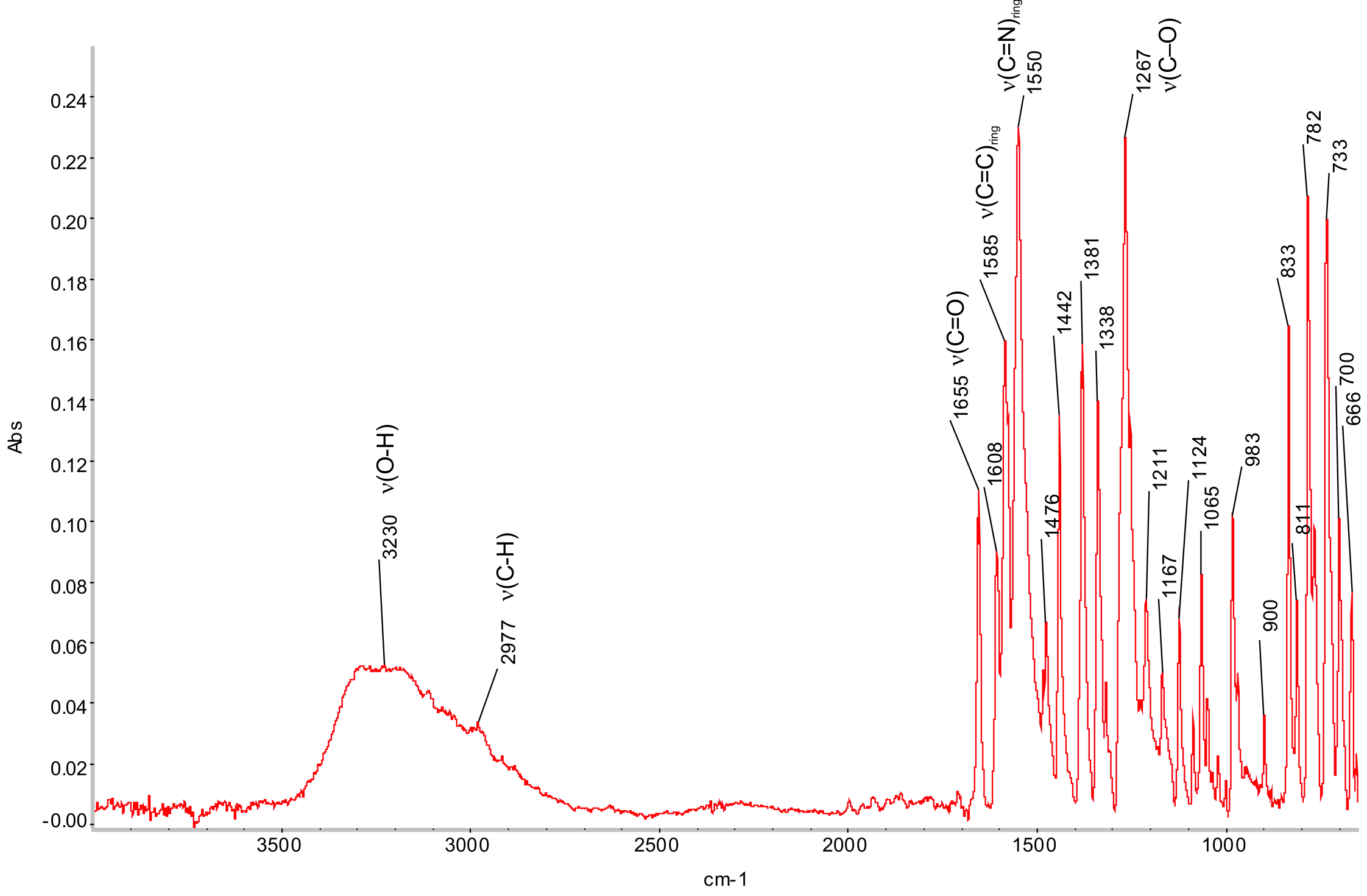

Supplement: S6 Fig — The maxima of the main peaks are noted. (TIF) [file pone.0181822.s007.tif]

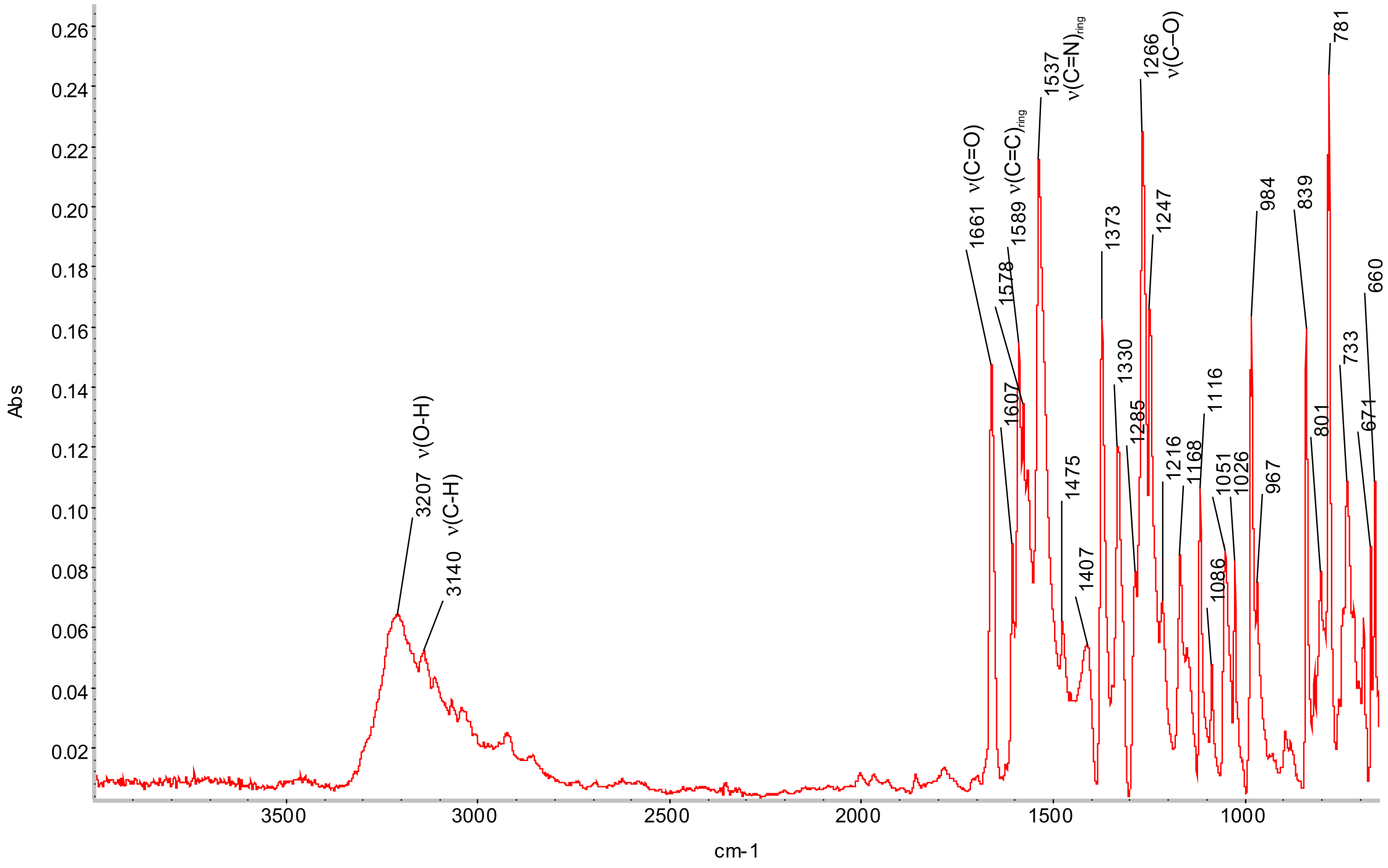

Supplement: S7 Fig — The maxima of the main peaks are noted. (TIF) [file pone.0181822.s008.tif]

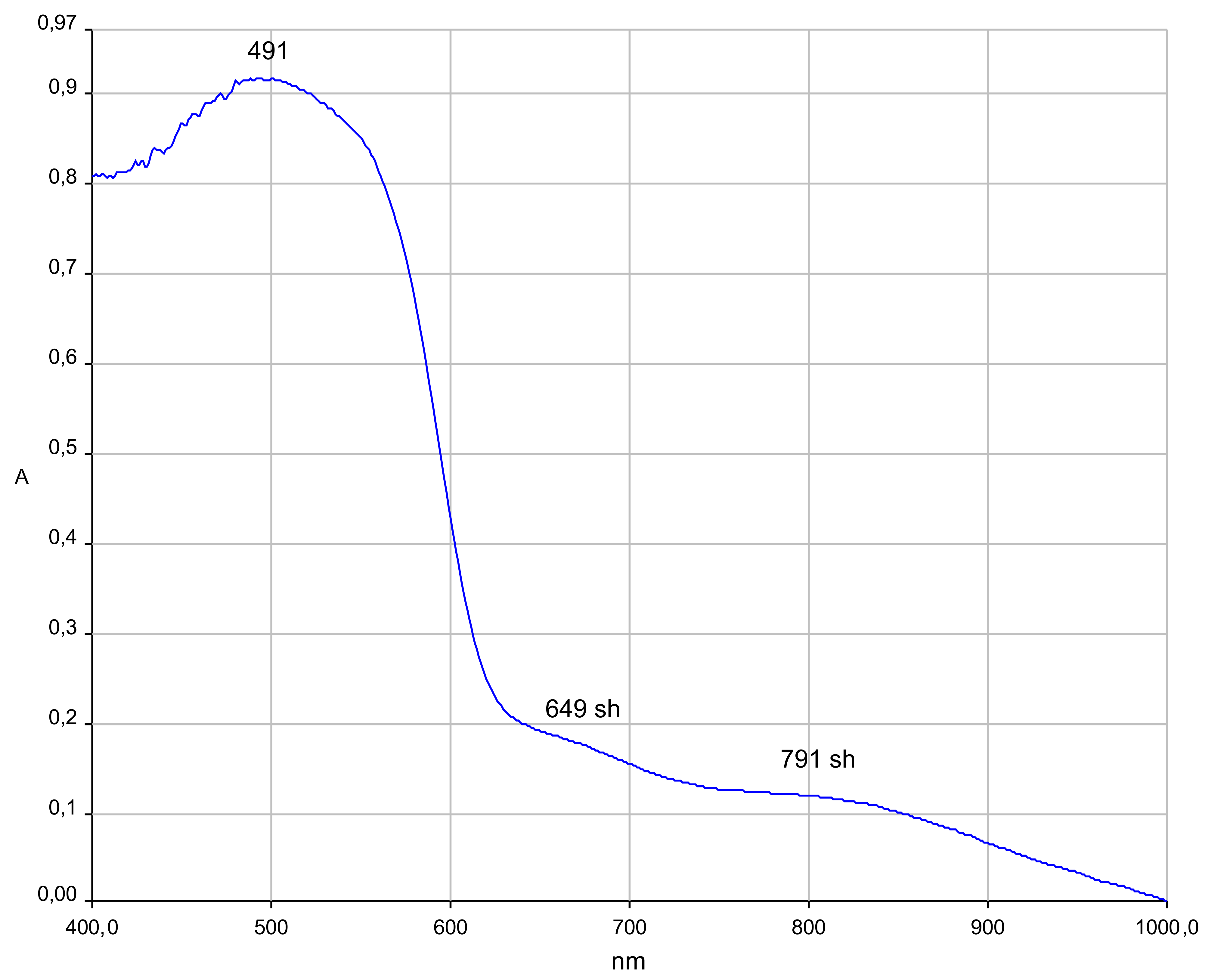

Supplement: S8 Fig — The remark sh means shoulder. (PNG) [file pone.0181822.s009.png]

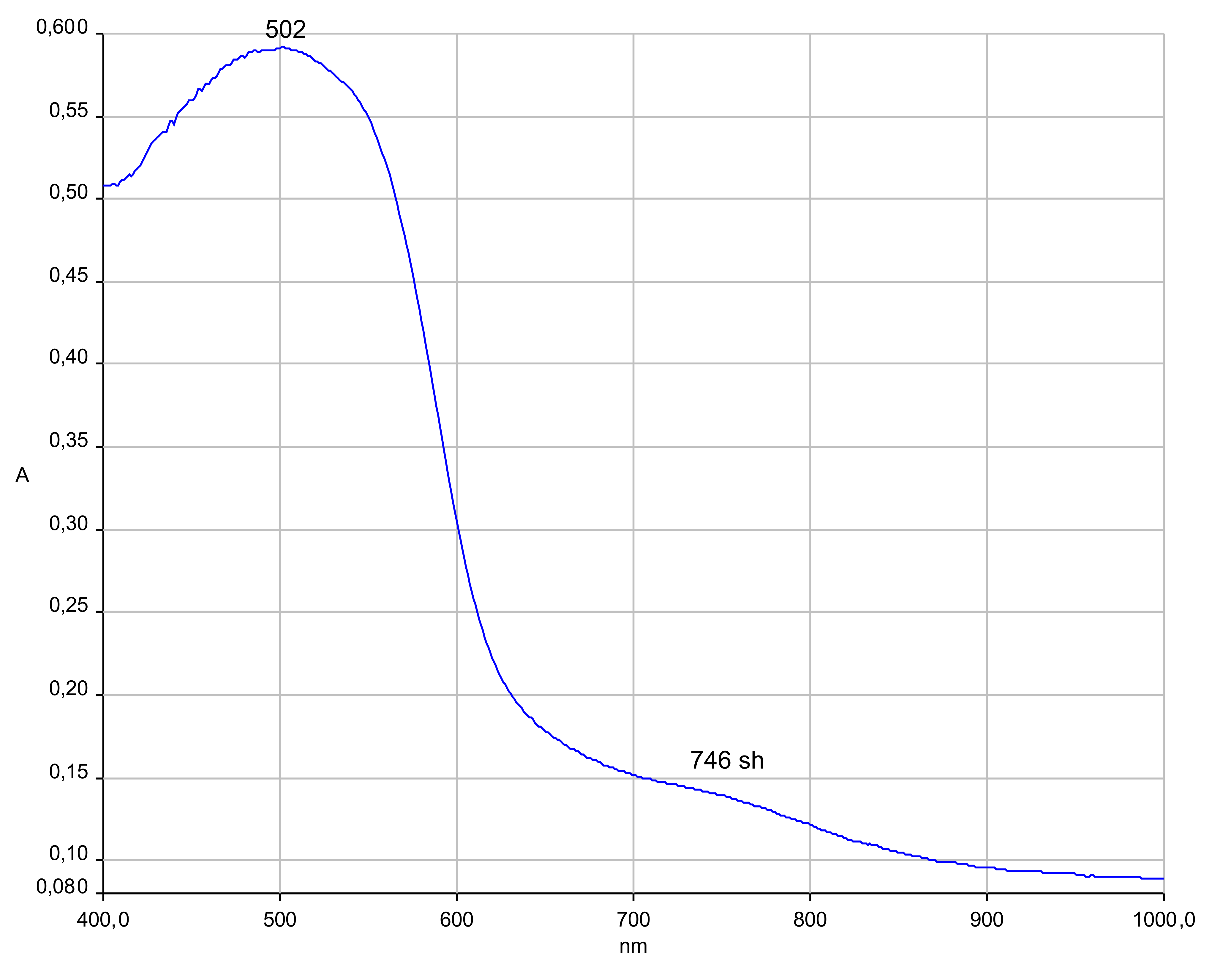

Supplement: S9 Fig — The remark sh means shoulder. (PNG) [file pone.0181822.s010.png]

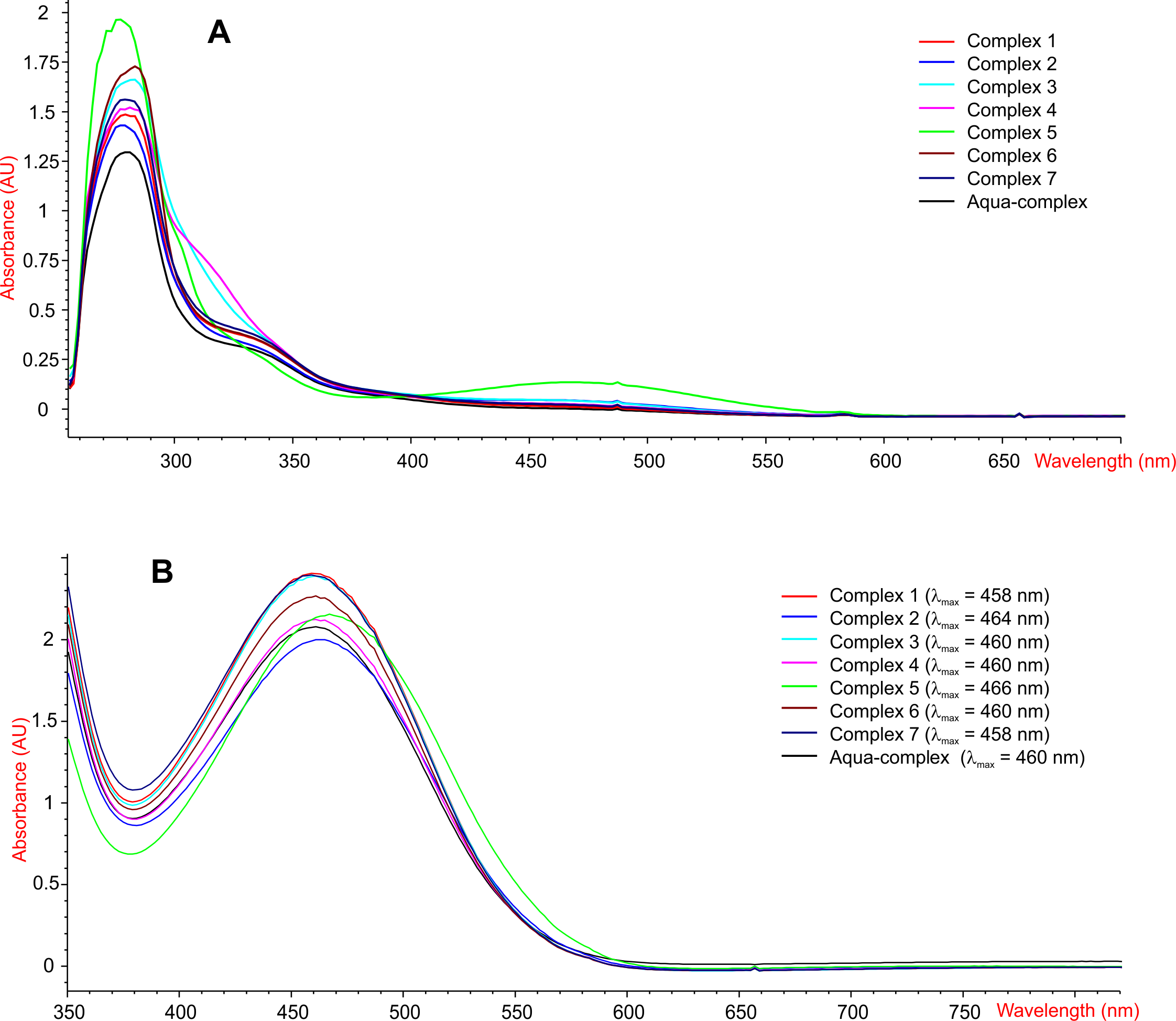

Supplement: S10 Fig — (TIF) [file pone.0181822.s011.tif]

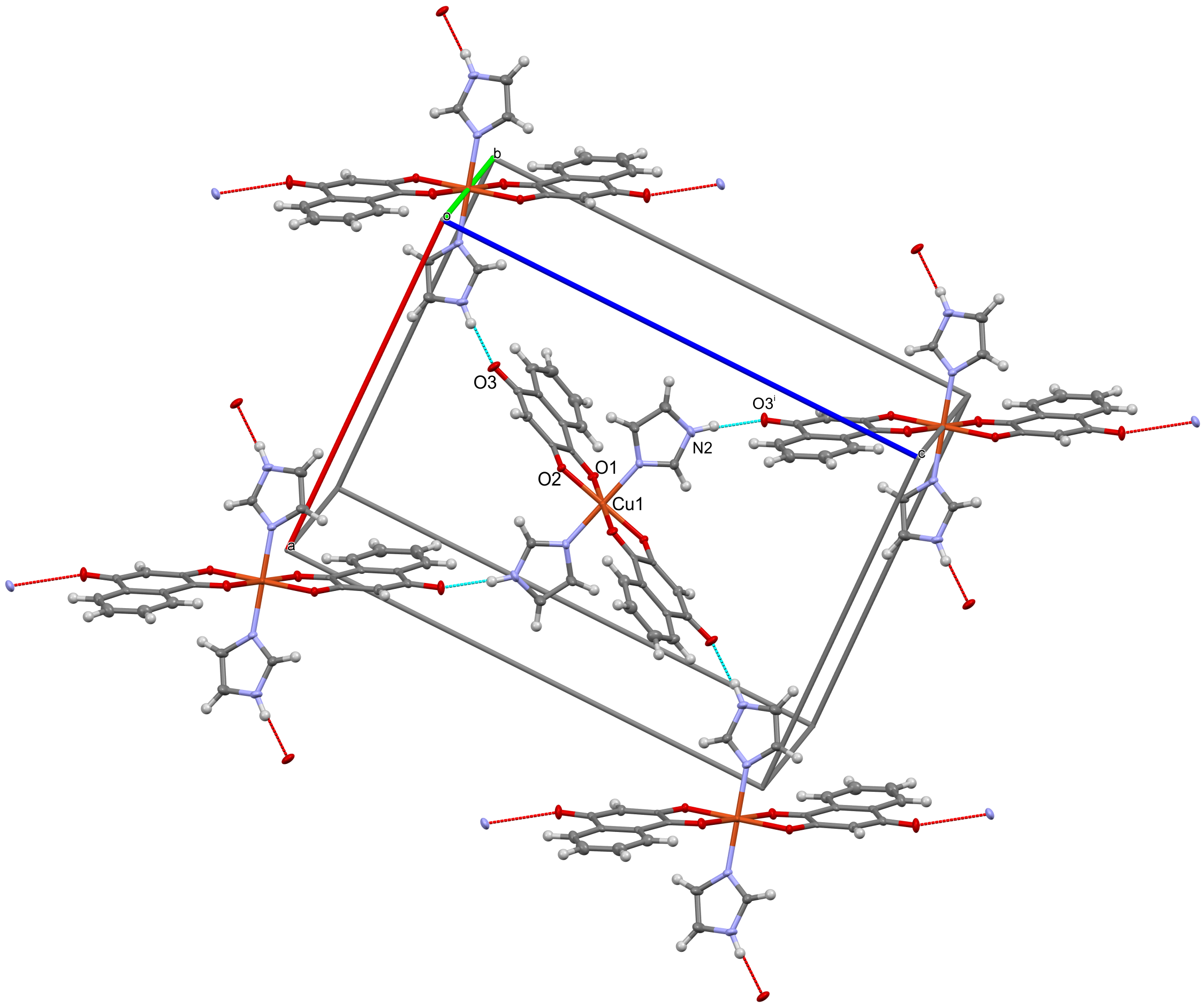

Supplement: S11 Fig — A distance d(N2…O3i) = 2.778(4) Å (symmetry code: (i) 1/2-x,1/2-y,1/2+z). (TIF) [file pone.0181822.s012.tif]

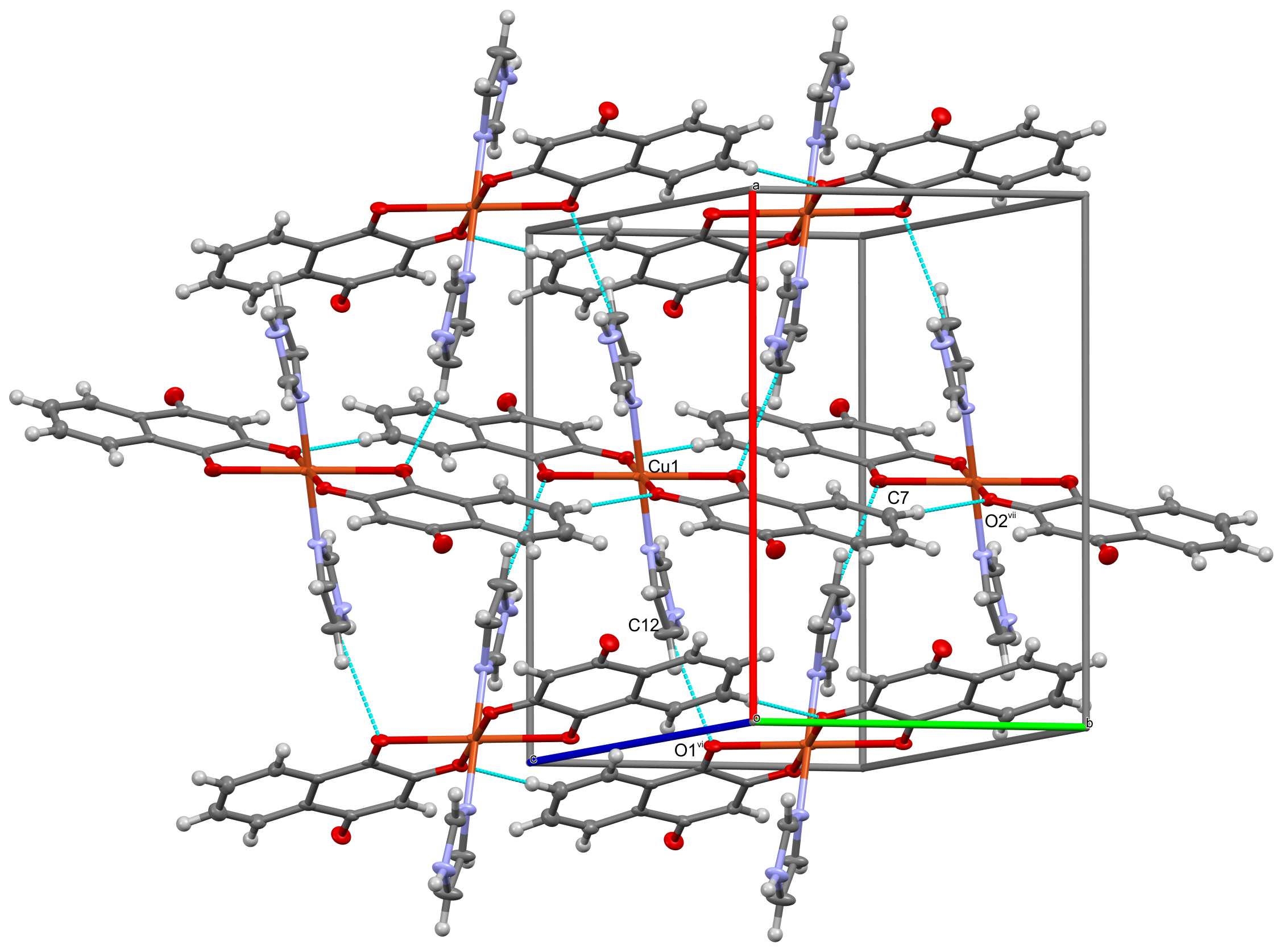

Supplement: S12 Fig — The distances of d(C12…O1i) = 3.199(5) Å and d(C7…O2ii) = 3.256(5) Å (symmetry codes: (vi) x-1/2,1/2-y,1-z; (vii) x,1+y,z). (TIF) [file pone.0181822.s013.tif]
