# Supplementary material for: In vitro and in vivo anti-inflammatory active copper(II)-lawsone complexes
Source: PLoS One. 2017 Jul 25;12(7):e0181822. doi: 10.1371/journal.pone.0181822 (PMC5526570; doi:10.1371/journal.pone.0181822)
Supplement: S1 Table — (PDF) [file pone.0181822.s014.pdf]

S1 Table. The interatomic parameters (in Å and °) of selected non-covalent interactions in the crystal structure of complex 2.

D–H···A	d(D-H)	d(H...A)	d(D...A)	<(DHA)
N2–H2a···O3^{i}	0.88	1.90	2.778(4)	174.3
C12–H12a···O1^{vi}	0.95	2.44	3.199(5)	136.4
C7–H7a···O2^{vii}	0.95	2.31	3.256(5)	174.2

Symmetry transformations used to generate equivalent atoms:
(i) 1/2-x,1/2-y,1/2+z; (vi) x-1/2,1/2-y,1-z; (vii) x,1+y,z)
